# Supplementary material for: Sae2 and Rif2 regulate MRX endonuclease activity at DNA double-strand breaks in opposite manners
Source: Cell Rep. 2021 Mar 30;34(13):108906. doi: 10.1016/j.celrep.2021.108906 (PMC8028314; doi:10.1016/j.celrep.2021.108906)
Supplement: Document S1. Figure S1 and Table S1 [file mmc1.pdf]

Cell Reports, Volume *34*

Supplemental information

Sae2 and Rif2 regulate MRX endonuclease activity at DNA double-strand breaks in opposite manners

Antonio Marsella, Elisa Gobbini, Corinne Cassani, Renata Tisi, Elda Cannavo, Giordano Reginato, Petr Cejka, and Maria Pia Longhese

SUPPLEMENTAL INFORMATION

Table S1, related to STAR methods. *Saccharomyces cerevisiae* strains used in this study.

Strain	Relevant genotype	Source
W303	*MAT*a/α *ade2-1 can1-100 his3-11,15 leu2-3,112 trp1-1 ura3-1 rad5-535*	
YLL 1134.2	W303 *rif2Δ::KANMX*	Cassani et al., 2016
DMP 6166/2D	W303 *MATα rif2Δ::HIS3 sae2Δ::KANMX*	This study
YLL 1069.3	W303 *sae2Δ::KANMX*	Cassani et al., 2018
HS21	*MATα ade5-1 his7-2 ura3Δ trp1-289 leu2-3,112::p305L3 LEU2 lys2::AluIR*	Lobachev et al., 2002
YLL 4197.1	HS21 *rif2Δ::TRP1*	This study
YLL 3773.3	HS21 *sae2Δ::KANMX*	This study
YLL 4348.1	HS21 *mre11-H15N::LEU2*	This study
DMP 7294/6A	HS21 *rif2Δ::TRP1 sae2Δ::KANMX*	This study
YLL 4349.2	HS21 *mre11-H15N::LEU2 rif2Δ::TRP1*	This study
JKM139	*MAT*a *hml*Δ::*ADE1*, *hmr*Δ::*ADE1*, *ade1-100, lys5, leu2-3,112, trp1::hisG ura3-52, ho, ade3::GAL-HO site*	Lee et al., 1998
YLL 1712.6	JKM139 *rif2Δ::HPHMX*	This study
DMP 5113/5A	JKM139 *KANMX::rad50-N18S*	This study
YLL 3627.3	JKM139 *sae2Δ::HPHMX*	This study
DMP 7144/8C	JKM139 *rif2Δ::KANMX sae2Δ::HPHMX*	This study
YLL 3757.1	JKM139 *KANMX::rad50-N18S sae2Δ::HPHMX*	This study
YLL 1854.2	JKM139 *MRE11-18MYC::TRP1*	Cassani et al., 2018
DMP 6149/2D	JKM139 *sae2Δ::KANMX MRE11-18MYC::TRP1*	This study
DMP 7248/3A	JKM139 *rif2Δ::KANMX sae2Δ::HPHMX MRE11-18MYC::TRP1*	This study
DMP 6211/5B	JKM139 *rif2Δ::HPHMX MRE11-18MYC::TRP1*	This study
YLL 3222.6	JKM139 *TEL1-3HA::NATMX*	Cassani et al., 2018
DMP 6435/1A	JKM139 *sae2Δ::KANMX TEL1-3HA::NATMX*	This study
DMP 7250/8D	JKM139 *sae2Δ::HPHMX rif2Δ::KANMX TEL1-3HA::NATMX*	This study
DMP 7250/8A	JKM139 *rif2Δ::KANMX TEL1-3HA::NATMX*	This study
YLL 3421.2	JKM139 *RAD9-3HA::TRP1*	This study
DMP 7192/6B	JKM139 *sae2Δ::HPHMX RAD9-3HA::TRP1*	This study
DMP 7192/8C	JKM139 *sae2Δ::HPHMX rif2Δ::KANMX RAD9-3HA::TRP1*	This study
DMP 7192/3A	JKM139 *rif2Δ::KANMX RAD9-3HA::TRP1*	This study
YLL 4104.2	JKM139 *MRE11-H125N-18MYC::TRP1*	This study

DMP 7283/3D	JKM139 *rif2Δ::KANMX MRE11-H125N-18MYC::TRP1*	This study
YLL 3754.1	W303 *KANMX::rad50-N18S sae2Δ::HIS3*	This study
DMP 7007/8B	W303 *KANMX::rad50-N18S sae2Δ::HIS3 rif2Δ::HIS3*	This study
DMP 6449/1C	W303 *KANMX::rad50-N18S*	This study
YLL 941.1	W303 *yku70Δ::HIS3*	This study
DMP 4374/6B	W303 *sae2Δ::KANMX yku70Δ::HIS3*	This study
DMP 6903/18D	W303 *KANMX::rad50-N18S ykuΔ::HIS3*	This study
DMP 6903/7D	W303 *KANMX::rad50-N18S sae2Δ::KANMX yku70Δ::HIS3*	This study
DMP 6903/9A	W303 *KANMX::rad50-N18S sae2Δ::KANMX yku70Δ::HIS3*	This study
DMP 7238/2A	JKM139 *KANMX::rad50-N18S MRE11-18MYC::TRP1*	This study
DMP 7238/7A	JKM139 *KANMX::rad50-N18S sae2Δ::HPHMX MRE11-18MYC::TRP1*	This study
DMP 7238/7A	JKM139 *KANMX::rad50-N18S sae2Δ::HPHMX rif2Δ::KANMX MRE11-18MYC::TRP1*	This study
YLL 3612.2	JKM139 *RIF2-18MYC::URA3*	This study
DMP 7059/2A	JKM139 *RIF2-18MYC::URA3 KANMX::rad50-N18S*	This study

Figure S1

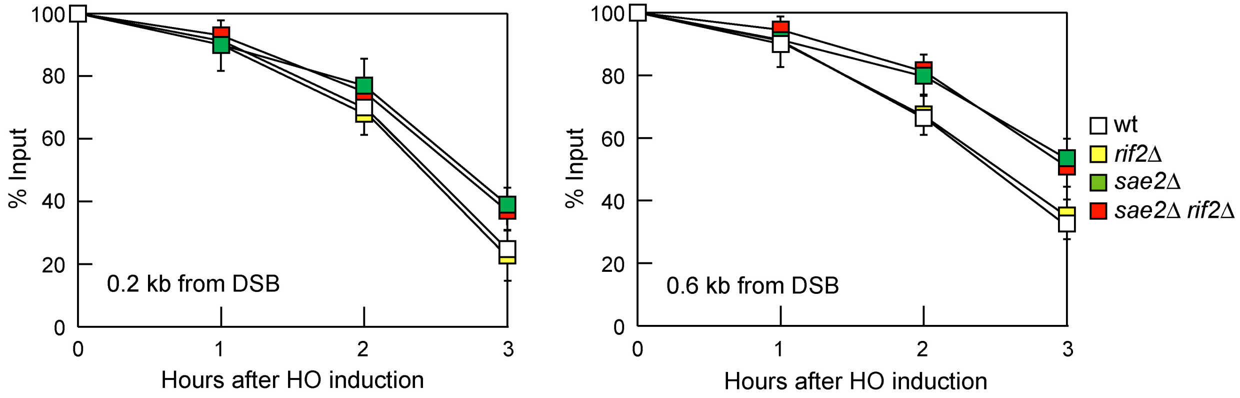

Figure S1, related to Figure 3A. Kinetics of input degradation at the HO-induced DSB. qPCR on input DNA at the indicated time points after HO induction (time zero). qPCR values at the HO-induced DSB were normalized to the corresponding values at the non-cleaved *ARO1* locus for each time points. Data are expressed relative to time zero that was set up at 100%. The mean values of three independent experiments are represented with error bars denoting s.d.
