# Supplementary material for: Sae2 and Rif2 regulate MRX endonuclease activity at DNA double-strand breaks in opposite manners
Source: Cell Rep. 2021 Mar 30;34(13):108906. doi: 10.1016/j.celrep.2021.108906 (PMC8028314; doi:10.1016/j.celrep.2021.108906)
Supplement: Document S2. Article plus supplemental information [file mmc2.pdf]

Cell Reports

Article

Sae2 and Rif2 regulate MRX endonuclease activity at DNA double-strand breaks in opposite manners

Graphical abstract

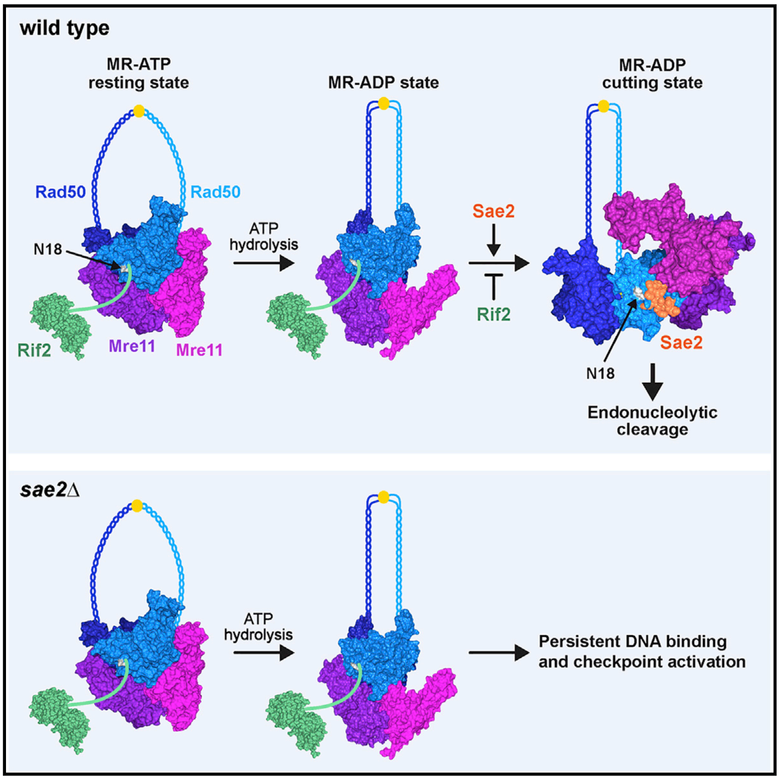

Highlights

- Sae2 stimulates Mre11 endonuclease activity by stabilizing the MRX cutting state
- Rif2 inhibits Sae2-mediated stimulation of Mre11 endonuclease activity
- The *rad50-N18S* mutation escapes Rif2-mediated inhibition of Mre11 nuclease
- Rif2 stabilizes an endonuclease inactive MR conformation that persistently binds DSBs

Authors

Antonio Marsella, Elisa Gobbini, Corinne Cassani, ..., Giordano Reginato, Petr Cejka, Maria Pia Longhese

Correspondence

mariapia.longhese@unimib.it

In brief

Phosphorylated Sae2 interacts with Rad50 and stimulates Mre11 endonuclease activity through an unknown mechanism. Marsella et al. show that Sae2 binding to the Rad50-Mre11 interface stabilizes a post-hydrolysis, endonuclease-active Mre11-Rad50 conformation. Rif2 interferes with the adoption of this conformation and inhibits Mre11 endonuclease activity.

Marsella et al., 2021, Cell Reports *34*, 108906
March 30, 2021 © 2021 The Author(s).
https://doi.org/10.1016/j.celrep.2021.108906

CellPress

Cell Reports

CellPress
OPEN ACCESS

Article

Sae2 and Rif2 regulate MRX endonuclease activity at DNA double-strand breaks in opposite manners

Antonio Marsella,[1] Elisa Gobbini,[1] Corinne Cassani,[1] Renata Tisi,[1] Elda Cannavo,[2] Giordano Reginato,[2,3] Petr Cejka,[2,3] and Maria Pia Longhese[1,4,*]

[1]Dipartimento di Biotecnologie e Bioscienze, Università degli Studi di Milano-Bicocca, Milano 20126, Italy
[2]Institute for Research in Biomedicine, Faculty of Biomedical Sciences, Università della Svizzera italiana (USI), Bellinzona, Switzerland
[3]Department of Biology, Institute of Biochemistry, Eidgenössische Technische Hochschule (ETH), Zürich, Switzerland
[4]Lead contact
*Correspondence: mariapia.longhese@unimib.it
https://doi.org/10.1016/j.celrep.2021.108906

SUMMARY

The Mre11-Rad50-Xrs2 (MRX) complex detects and processes DNA double-strand breaks (DSBs). Its DNA binding and processing activities are regulated by transitions between an ATP-bound state and a post-hydrolysis cutting state that is nucleolytically active. Mre11 endonuclease activity is stimulated by Sae2, whose lack increases MRX persistence at DSBs and checkpoint activation. Here we show that the Rif2 protein inhibits Mre11 endonuclease activity and is responsible for the increased MRX retention at DSBs in *sae2Δ* cells. We identify a Rad50 residue that is important for Rad50-Rif2 interaction and Rif2 inhibition of Mre11 nuclease. This residue is located near a Rad50 surface that binds Sae2 and is important in stabilizing the Mre11-Rad50 (MR) interaction in the cutting state. We propose that Sae2 stimulates Mre11 endonuclease activity by stabilizing a post-hydrolysis MR conformation that is competent for DNA cleavage, whereas Rif2 antagonizes this Sae2 function and stabilizes an endonuclease inactive MR conformation.

INTRODUCTION

DNA double-strand breaks (DSBs) are highly cytotoxic lesions that can be repaired by non-homologous end joining (NHEJ) or homologous recombination (HR) (Mehta and Haber, 2014). Although NHEJ involves the direct ligation of the DSB ends, HR uses the DNA information stored in a homologous duplex DNA as a template for repair. The obligate step of HR is the degradation of the 5′-terminated DNA strands at both DSB ends to generate 3′ end single-stranded DNA (ssDNA) overhangs that catalyze homologous pairing and strand invasion (Ranjha et al., 2018).

Generation of DNA DSBs triggers activation of a checkpoint response that couples DSB repair with cell-cycle progression (Villa et al., 2016). Apical checkpoint proteins include Mec1 and Tel1 kinases (ATR and ATM in mammals, respectively). Once activated by damaged DNA, Tel1 and Mec1 transduce the checkpoint signals to the downstream effector kinases Rad53 and Chk1 (CHK2 and CHK1 in mammals, respectively), whose activation requires the conserved protein Rad9 (53BP1 in mammals) (Gilbert et al., 2001; Sweeney et al., 2005; Schwartz et al., 2002).

The evolutionarily conserved Mre11-Rad50-Xrs2/NBS1 complex (MRX in *Saccharomyces cerevisiae* and MRN in mammals, *Schizosaccharomyces pombe*, and *Xenopus laevis*) plays multiple functions at DSBs. It tethers DNA ends, initiates resection of the DSB ends, and signals the presence of DSBs by recruiting and activating Tel1/ATM (Syed and Tainer, 2018). The Sae2 protein (CtIP in mammals) activates a latent Mre11 endonuclease activity within the context of the MRX complex to incise the 5′-terminated strands at the double-stranded DNA (dsDNA) ends (Cannavo and Cejka, 2014). This function requires Sae2/CtIP phosphorylation by cyclin-dependent kinases (CDKs) (Cannavo and Cejka, 2014; Anand et al., 2016; Cannavo et al., 2018). Then, the Mre11 exonuclease degrades DNA in the 3′-5′ direction toward the DSB ends, whereas the nuclease Exo1 or Dna2 processes the 5′-terminated strands (Mimitou and Symington, 2008; Zhu et al., 2008; Cejka et al., 2010; Niu et al., 2010; Garcia et al., 2011; Shibata et al., 2014; Reginato et al., 2017; Wang et al., 2017). The initial 5′ DNA end resection catalyzed by MRX is important for the processing of DSB ends carrying non-canonical structures, such as protein blocks or secondary DNA structures (Reginato and Cejka, 2020). This has been demonstrated in meiosis, in which Sae2 and the Mre11 endonuclease activity are required to resect DSBs covalently bound by Spo11, a topoisomerase-like protein that generates DSBs by forming a covalent linkage between a conserved tyrosine residue and the 5′ end of the cleaved strand (Bergerat et al., 1997; Keeney et al., 1997; Moreau et al., 1999; Neale et al., 2005).

At the molecular level, the core Mre11-Rad50 (MR) complex exists as a hetero-tetrameric assembly (M_2R_2), in which Mre11 dimerizes and interacts with Rad50 (Syed and Tainer, 2018; Tisi et al., 2020). Structural studies have shown that MR possesses a globular head, derived from the association of two Rad50 nucleotide binding domains (NBDs) with two Mre11 nuclease domains, and a long projection constituted by anti-parallel coiled-coil

This is an open access article under the CC BY license (http://creativecommons.org/licenses/by/4.0/).

domains that are joined by a Zn-hook dimerization motif (Hopfner et al., 2001, 2002; Moreno-Herrero et al., 2005; Park et al., 2017).

Rad50 possesses ATPase motifs at the N and C termini. Structural studies have shown that ATP binding to Rad50 engages the two Rad50 NBDs within the catalytic head, leading to a higher-affinity DNA binding conformation (Hopfner et al., 2000; Williams et al., 2008, 2011; Lammens et al., 2011; Lim et al., 2011; Möckel et al., 2012; Liu et al., 2016; Seifert et al., 2016). In the ATP-bound conformation, Rad50 blocks Mre11 access to dsDNA and therefore its nuclease activity. Rad50-catalyzed ATP hydrolysis triggers large conformational changes, leading to a dislocation of Rad50 that makes DNA accessible to the Mre11 nuclease active sites (Lammens et al., 2011; Williams et al., 2011; Möckel et al., 2012; Deshpande et al., 2014). A recent cryo-electron microscopy (cryo-EM) study of the *Escherichia coli* SbcC-SbcD complex (orthologs of MR) shows that Mre11 and Rad50 in the post-ATP hydrolysis cutting configuration share an interface that involves an outer β sheet of Rad50 and an Mre11 loop, defined as a fastener (Käshammer et al., 2019). This interface is essential for the nuclease activity of the complex, because mutants losing this interaction, such as Rad50-E115K or Mre11-K149E, are nuclease defective (Käshammer et al., 2019). Although the fastener loop is not conserved, we propose that an analog of the fastener exists in the eukaryotic MRX/MRN complex as well.

Sae2 not only stimulates the endonuclease activity of the MRX complex but also controls MRX persistence to DSBs. The lack of Sae2 increases MRX accumulation at the DSB ends, which leads to enhanced Tel1 signaling activity (Usui et al., 2001; Lisby et al., 2004; Clerici et al., 2006). *mre11* nuclease-dead mutants also exhibit persistent MRX and Tel1 association at DSB ends (Lisby et al., 2004; Yu et al., 2018), suggesting that MRX-Sae2 cleavage activity contributes to regulate MRX association to DNA ends. This persistent MRX-Tel1 activation in *sae2*Δ cells is associated with enhanced Rad9 association at DSBs and Rad53 activation, which increases the DNA damage sensitivity of *sae2*Δ cells by causing permanent cell-cycle arrest (Usui et al., 2001; Clerici et al., 2006; Yu et al., 2018). Mutations that decrease MRX association to DSBs restore DNA damage resistance in Sae2-deficient cells (Chen et al., 2015; Puddu et al., 2015; Cassani et al., 2018). A similar effect occurs when Tel1 or Rad53 function is affected by reducing either their kinase activity or Rad9-Rad53 interaction (Ferrari et al., 2015; Gobbini et al., 2015; Yu et al., 2018).

In *S. cerevisiae*, Rad50 is known to interact with Rif2, which is recruited to telomeric DNA ends and negatively regulates telomerase-mediated telomere elongation (Wotton and Shore, 1997; Levy and Blackburn, 2004; Hirano et al., 2009; Martina et al., 2012). It has been shown that Rif2 reduces the association of MRX and Tel1 at telomeres (Hirano et al., 2009; Cassani et al., 2016). Tel1, whose recruitment to both DSBs and telomeres requires its interaction with the C terminus of Xrs2, plays a structural role in stabilizing MRX association to DSBs (Cassani et al., 2016). Because Rif2 interacts with Xrs2 within the same C-terminal region as Tel1, it has been proposed that Rif2 counteracts MRX persistence at DSBs by competing with Tel1 for Xrs2 binding (Hirano et al., 2009). However, Rif2 inhibits MRX-dependent stimulation of Tel1 kinase activity independently of Xrs2 (Hailemariam et al., 2019). Furthermore, Rif2 interacts *in vitro* with Rad50 and stimulates its ATPase activity in a Xrs2-independent manner (Cassani et al., 2016; Hailemariam et al., 2019), suggesting that Rif2 acts directly on Rad50 to control MRX and Tel1 activity. This Rif2-mediated inhibition of Tel1 relies on the BAT (blocks addition of telomeres) motif (Hailemariam et al., 2019), which is localized at the extreme Rif2 N-terminal region and has been implicated in telomere length regulation (Kaizer et al., 2015).

Here we show that Rif2 inhibits Sae2-mediated stimulation of MR endonuclease activity. Furthermore, Rif2 is responsible for the increased MRX and Tel1 association at DSBs in *sae2*Δ cells. The *rad50-N18S* mutation, which weakens Rad50-Rif2 interaction, partially escapes both Rif2-mediated inhibition of the Mre11 nuclease and Rif2-mediated stabilization of MRX association at DSBs. This mutation is located near a Rad50 surface that binds Sae2 and is important in stabilizing the interaction between Mre11 and Rad50 NBDs in the cutting state (Käshammer et al., 2019). These findings, together with structural studies, lead to a model whereby Sae2 stimulates Mre11 endonuclease activity by stabilizing the MRX cutting state, whereas Rif2 counteracts this Sae2 function and stabilizes an MRX conformation that can bind DNA ends but is not competent to cleave them.

RESULTS

The lack of Rif2 suppresses the DNA damage hypersensitivity of *sae2*Δ cells

Phosphorylated Sae2 (pSae2) stimulates the endonuclease of Mre11 within the MRX complex (Cannavo and Cejka, 2014). Although Sae2 does not appear to influence the overall Rad50 ATP hydrolysis rate, stimulation of Mre11 nuclease by Sae2 requires ATP hydrolysis by Rad50 (Cannavo et al., 2019), suggesting that Sae2 helps couple ATP hydrolysis by Rad50 with productive endonucleolytic DNA cleavage by Mre11.

Because Rif2 has been shown to stimulate Rad50 ATPase activity (Cassani et al., 2016; Hailemariam et al., 2019), to better understand the interplay between Sae2 and Rif2 in MRX regulation, we analyzed the effect of deleting *RIF2* from *sae2*Δ cells. *sae2*Δ *rif2*Δ double-mutant cells were more resistant to camptothecin (CPT) and methyl methanesulfonate (MMS) compared with *sae2*Δ cells (Figure 1A), indicating that the lack of Rif2 partially suppresses the DNA damage sensitivity of *sae2*Δ cells.

The Sae2-induced MR endonuclease is essential to resect meiotic DSBs and to cleave DNA hairpin structures (Trujillo and Sung, 2001; Lobachev et al., 2002; Neale et al., 2005; Garcia et al., 2011). The topoisomerase-like protein Spo11 initiates meiotic recombination by cleaving both DNA strands at recombination hotspots and remains covalently bound to the 5′ end of the cleaved strand (Keeney et al., 1997). Removal of Spo11 is essential for subsequent steps of HR and spore formation, and it is catalyzed by Mre11 endonuclease and Sae2 (Neale et al., 2005; Garcia et al., 2011). As expected, both *sae2*Δ and nuclease-defective *mre11-H125N* (hereafter called *mre11-nd*) diploid cells failed to sporulate, consistent with impaired Spo11 removal (Figure 1B) (McKee and Kleckner, 1997; Prinz et al., 1997; Usui et al., 1998). By contrast, the percentage of sporulation was not affected by *RIF2* deletion, which failed to suppress the sporulation defect of *sae2*Δ and *mre11-nd* cells

CellPress
OPEN ACCESS

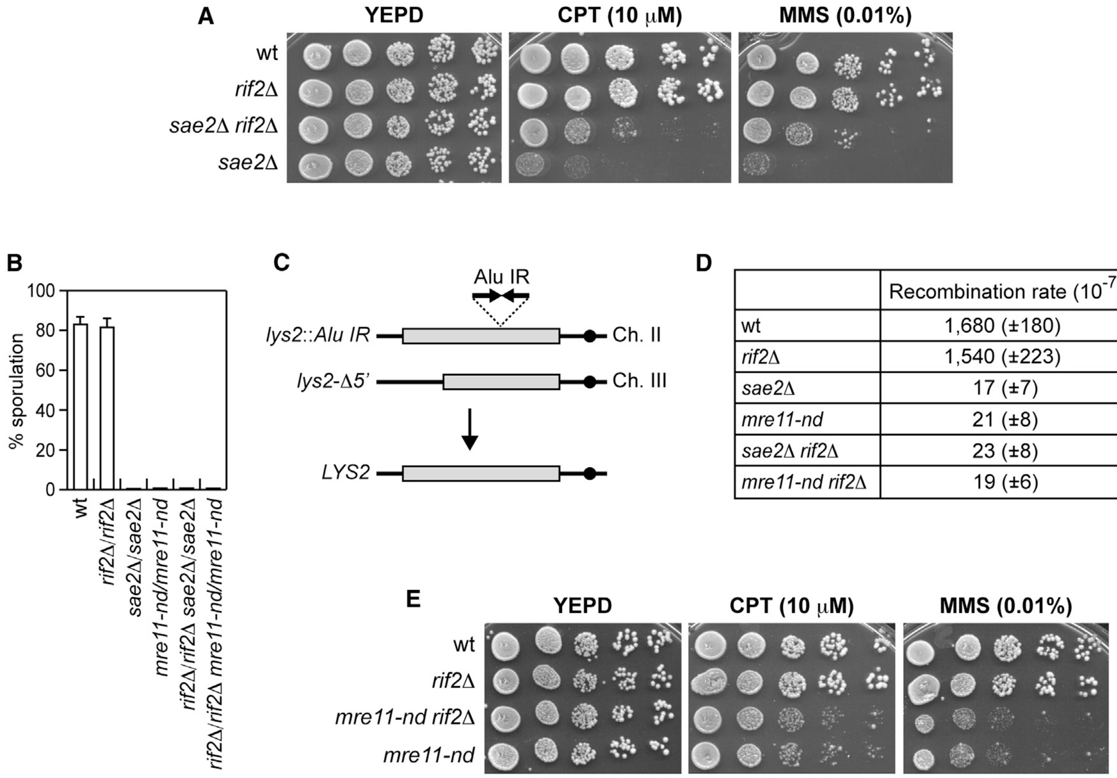

Figure 1. The lack of Rif2 partially restores DNA damage resistance of *sae2Δ* cells

(A) Exponentially growing cultures were serially diluted (1:10), and each dilution was spotted out onto yeast extract, bactopeptone, and glucose (YEPD) plates with or without CPT or MMS.

(B) Percentage of sporulation. Diploid cells homozygous for the indicated mutations were induced to enter in meiosis. The reported values are the mean values of 3 independent experiments, with error bars denoting SD.

(C) Schematic representation of the *lys2-Alu IR* and *lys2-Δ5′* ectopic recombination system.

(D) Recombination frequency of strains with the *lys2-Alu IR* and *lys2-Δ5′* ectopic recombination system. The rate of Lys+ recombinants was derived from the median recombination frequency. The reported values are the mean values of 3 independent experiments, with SD indicated in brackets.

(E) Exponentially growing cultures were serially diluted (1:10), and each dilution was spotted out onto YEPD plates with or without CPT or MMS.

(Figure 1B). This finding suggests that loss of Rif2 does not restore Sae2 function in resecting meiotic DSBs.

Hairpin cleavage was assessed using a previously described genetic assay (Lobachev et al., 2002), in which inverted Alu elements inserted in the *LYS2* gene on chromosome II stimulate ectopic recombination with a truncated *lys2* copy (*lys2-Δ5′*) located on chromosome III to generate Lys+ prototrophs (Figure 1C). This recombination largely depends on Mre11 endonuclease and Sae2 (Lobachev et al., 2002). The inverted Alu repeats are thought to extrude to form a hairpin or cruciform structure that is cleaved by an unknown nuclease to form a hairpin-capped DSB, which is then cleaved by MRX-Sae2 and subsequently repaired by HR (Lobachev et al., 2002). *RIF2* deletion failed to suppress the hairpin resolution defect conferred by either the *SAE2* deletion or the *mre11-nd* allele (Figure 1D).

Altogether, these findings indicate that *RIF2* deletion does not suppress *sae2Δ* DNA damage sensitivity by activating the Mre11 nuclease independently of Sae2. Consistent with this conclusion, *RIF2* deletion failed to suppress the DNA damage hypersensitivity of *mre11-nd* cells (Figure 1E).

The lack of Rif2 dampens checkpoint signaling in *sae2Δ* cells by decreasing MRX, Tel1, and Rad9 association to DSBs

Previous studies demonstrated that the increased DNA damage sensitivity of *sae2Δ* cells partly results from increased retention of MRX, Tel1, and Rad9 at DSBs, which leads to persistent Rad53 phosphorylation and activation (Usui et al., 2001; Lisby et al., 2004; Clerici et al., 2006). Thus, we asked whether *RIF2* deletion can partially suppress the DNA damage sensitivity of *sae2Δ* cells by decreasing checkpoint activation. To measure checkpoint activation, we used a haploid strain carrying the *HO* gene under the control of a galactose-inducible promoter (Lee et al., 1998). In this strain, induction of HO by galactose addition leads to generation at the *MAT* locus of a single DSB that cannot be repaired by HR because of the lack of the homologous donor loci *HML* and *HMR*. Checkpoint activation was monitored by following cell-cycle progression and Rad53 phosphorylation, which is required for Rad53 activation and is detectable as a decrease of its electrophoretic mobility. When wild-type, *rif2Δ*, *sae2Δ*, and *sae2Δ rif2Δ* cells were spotted on galactose-containing plates to induce HO, all cell cultures arrested at the 2-cell dumbbell stage

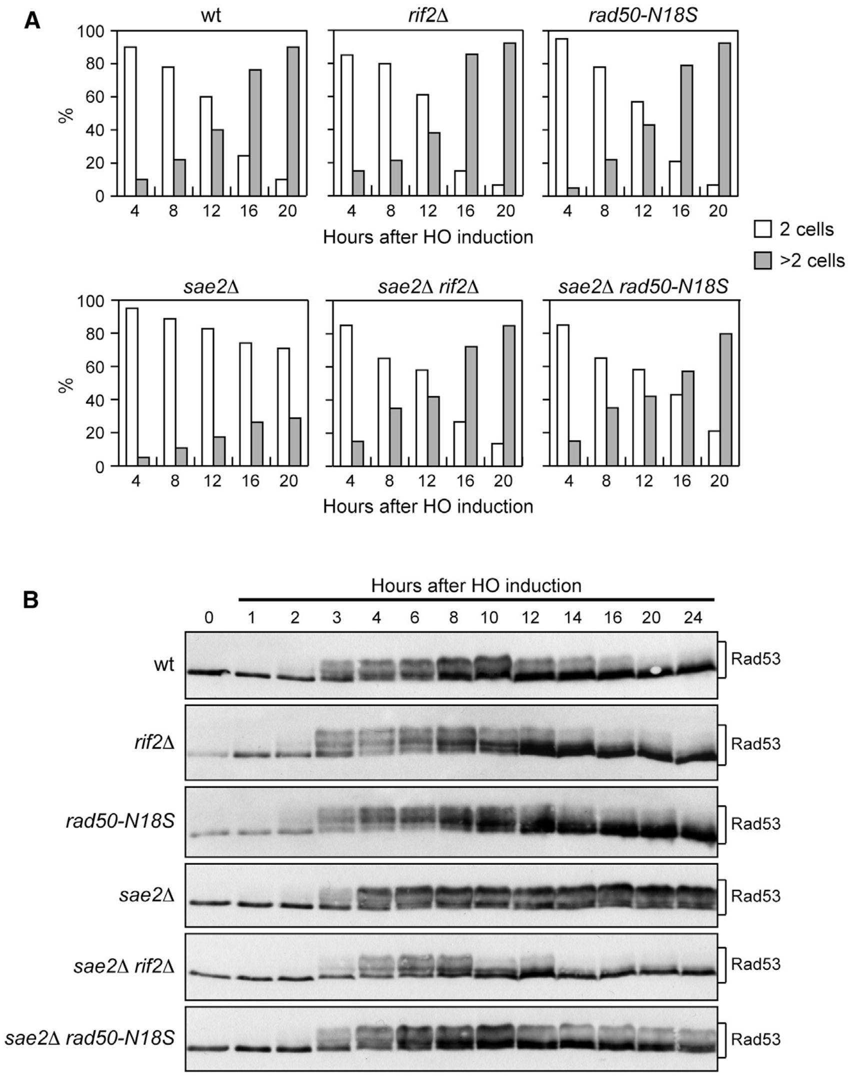

Figure 2. The *rif2*Δ and *rad50-N18S* alleles decreases checkpoint activation in *sae2*Δ cells

(A) Yeast extract, bactopeptone, and raffinose (YEPR) G1-arrested cell cultures were plated on galactose-containing plates (time zero). At the indicated time points, 200 cells for each strain were analyzed to determine the frequency of large budded cells (2 cells) and of cells forming microcolonies of more than two cells (>2 cells). This experiment was repeated three times with similar results.

(B) Rad53 phosphorylation after a HO-induced DSB. Exponentially growing YEPR cultures were transferred to yeast extract, bactopeptone, raffinose, and galactose (YEPRG) (time zero) to induce HO expression, followed by western blot analysis with anti-Rad53 antibodies.

(Figure 2A) and phosphorylated Rad53 (Figure 2B) about 3–4 h after HO induction. Consistent with previous data (Clerici et al., 2006), *sae2*Δ cells increased the amount of the slowest migrating Rad53 phosphorylated form compared with wild-type cells after HO induction (Figure 2B). *RIF2* deletion, which did not affect Rad53 phosphorylation by itself, decreased the amount of HO-induced Rad53 phosphorylation in *sae2*Δ cells to almost wild-type levels (Figure 2B).

A checkpoint response triggered by a single unrepairable DSB eventually can be turned off, allowing cells to resume cell-cycle progression through a process called adaptation (Usui et al., 2001; Lisby et al., 2004; Clerici et al., 2006). The increased Tel1-mediated Rad53 activation in *sae2*Δ cells prevents cells from adapting to an unrepaired DSB (Clerici et al., 2006). Rad53 phosphorylation decreased in wild-type and *rif2*Δ cells 12 h after galactose addition, when cells started to form colonies with more than two cells; however, as expected, it persisted longer in *sae2*Δ cells that remained arrested at the 2-cell dumbbell stage for at least 20 h (Figures 2A and 2B). Remarkably, the amount of phosphorylated Rad53 decreased 12 h after galactose addition in *sae2*Δ *rif2*Δ cells (Figure 2B), which formed microcolonies with more than two cells with almost wild-type kinetics (Figure 2A). Thus, we can conclude that *RIF2* deletion decreases Tel1-mediated checkpoint activation of *sae2*Δ cells.

MRX is required to recruit and activate Tel1, which in turn transduces the checkpoint signals to Rad53 through the Rad9 protein (Usui et al., 2001; Gilbert et al., 2001; Sweeney et al., 2005; Schwartz et al., 2002). Previous works have established that *mre11* alleles that reduce MRX binding to DSBs restore DNA damage resistance of *sae2*Δ cells by decreasing Tel1-dependent Rad53 activation (Chen et al., 2015; Puddu et al., 2015; Cassani et al., 2018). Thus, we measured Mre11, Tel1, and Rad9 association to the HO-induced DSB by chromatin immunoprecipitation (ChIP) and quantitative PCR (qPCR). The lack of Rif2 decreased the high amount of Mre11, Tel1, and Rad9 bound to the HO-induced DSB in *sae2*Δ cells (Figure 3A), thus explaining the diminished Rad53 activation in *sae2*Δ *rif2*Δ cells compared with *sae2*Δ cells. The decreased Mre11 association at the HO-induced DSB in *sae2*Δ *rif2*Δ cells compared with *sae2*Δ cells did not result from either diminished Mre11 levels or increased degradation of the DSB ends. Protein extracts from *sae2*Δ and *sae2*Δ *rif2*Δ cells contained similar amounts of Mre11 protein (Figure 3B). Furthermore, *RIF2* deletion did not alter the degradation kinetics of the DSB ends either in the presence or in the absence of Sae2 (Figure S1). This finding indicates that Rif2 is responsible for the stabilization of the MRX complex at DSBs in *sae2*Δ cells, which in turn leads to unscheduled checkpoint activation.

Abrogation of Mre11 nuclease activity increases the amount of MRX and therefore of Tel1 bound at DSBs to an extent similar to

CellPress
OPEN ACCESS

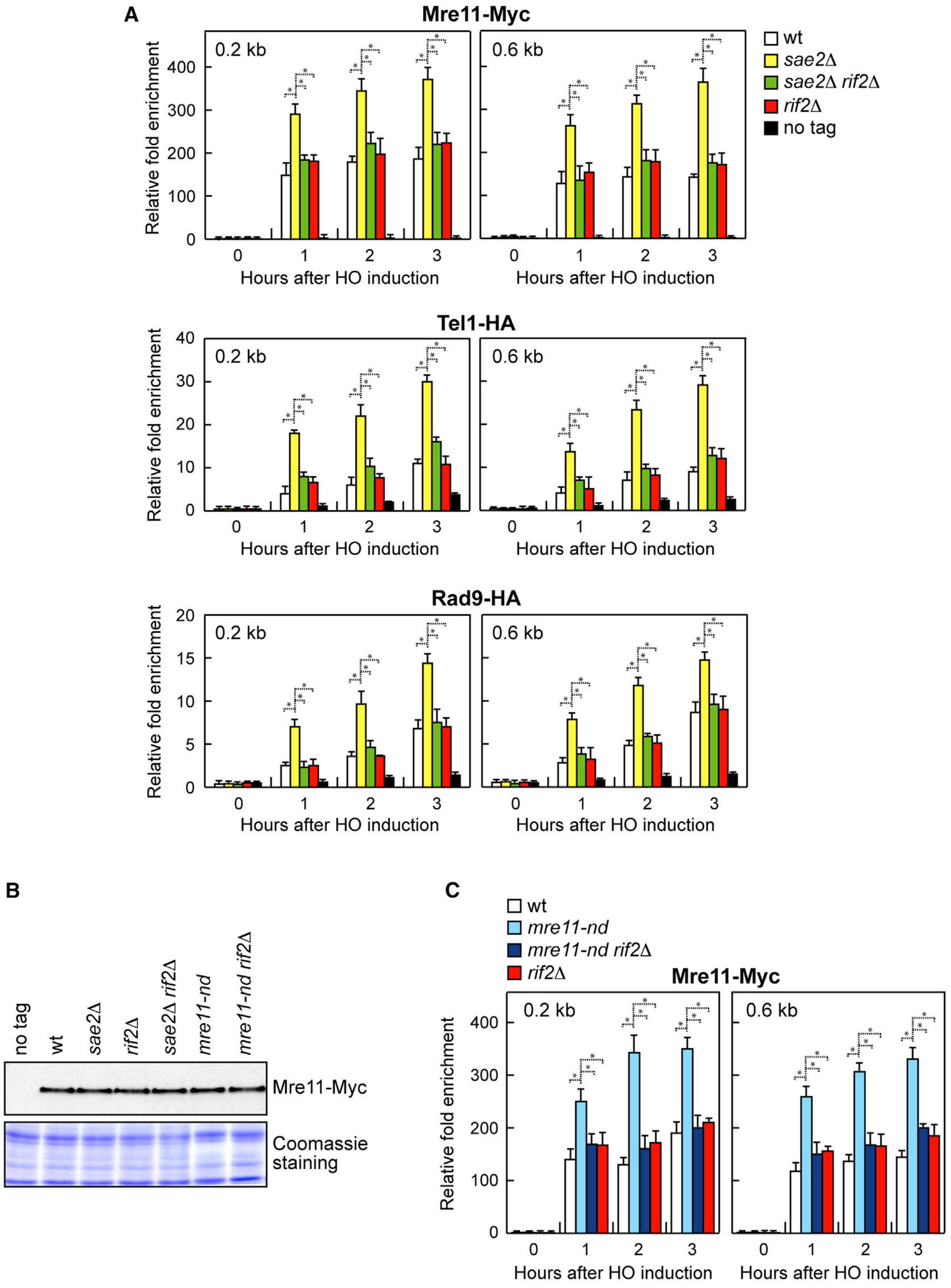

Figure 3. The lack of Rif2 decreases MRX abundance at DSBs in both *sae2Δ* and *mre11-nd* cells

(A) ChIP and qPCR. Exponentially growing YEPR cell cultures were transferred to YEPRG to induce HO expression, followed by ChIP analysis of the recruitment of the indicated proteins at the indicated distance from the HO-cut site. In all diagrams, ChIP signals were normalized for each time point to the corresponding input signal. The mean values of three independent experiments are represented with error bars denoting SD. *$p < 0.005$ (Student's t test).

(B) Western blot with anti-Myc antibodies of extracts used for the ChIP analysis shown in (A). The same amount of protein extracts was separated on SDS-PAGE and stained with Coomassie blue as loading control.

(C) ChIP and qPCR. Exponentially growing YEPR cell cultures were transferred to YEPRG to induce HO expression, followed by ChIP analysis of the recruitment of Mre11-Myc as described in (A). The mean values of three independent experiments are represented with error bars denoting SD. *$p < 0.005$ (Student's t test).

that caused by *SAE2* deletion (Lisby et al., 2004; Yu et al., 2018), suggesting that MRX-Sae2-mediated cleavage activity contributes to decrease MRX persistence at DSBs. Thus, we asked whether the lack of Rif2 reduces the association to DSBs of the Mre11-nd variant. The amount of Mre11-nd bound at the HO-induced DSB was higher in the presence than in the absence of Rif2 (Figure 3C), indicating that Rif2 is responsible for the increased persistence at DSBs of nuclease-defective MRX. Rif2 increases MRX retention at DSBs only when Sae2 is absent or Mre11 is catalytically inactive. The *rif2*Δ cells did not show a reduced Mre11 and Tel1 association at the HO-induced DSB (Figure 3A). Rather, and consistent with previous data (Cassani et al., 2016), the lack of Rif2 by itself leads to a slight increased Mre11 and Tel1 persistence at DSBs (Figures 3A and 3C).

Although Rif2 is responsible for enhanced association to DSBs of nuclease-defective Mre11-nd, *RIF2* deletion was unable to suppress the DNA damage sensitivity of *mre11-nd* mutant cells (Figure 1E) (Yu et al., 2018). However, in contrast to *sae2*Δ cells, *mre11-nd* cells, which are considerably more resistant to DNA-damaging agents than *sae2*Δ cells, do not enhance Rad9 accumulation at DSBs and checkpoint activation (Yu et al., 2018; Colombo et al., 2019), supporting the conclusion the *RIF2* deletion partially restores the DNA damage resistance of *sae2*Δ cells by dampening checkpoint signaling.

Rad50-N18S mimics *RIF2* deletion with respect to *sae2*Δ suppression and checkpoint inhibition

The MR complex was shown to efficiently bind dsDNA in presence of ATP-bound Rad50, which generates a groove that can host dsDNA (Lammens et al., 2011; Rojowska et al., 2014; Liu et al., 2016; Seifert et al., 2016). The finding that Rif2 increases ATPase activity by Rad50 (Cassani et al., 2016; Hailemariam et al., 2019) suggests that Rif2 can regulate MRX persistence at DSBs by acting on the Rad50 subunit. Thus, to better understand the interplay between Rif2 and Rad50, we searched for *rad50* mutants that are insensitive to Rif2 regulation. Because *RIF2* deletion suppresses the DNA damage sensitivity of *sae2*Δ cells, we screened for *rad50* alleles that restored the DNA damage resistance of *sae2*Δ cells. *RAD50* gene was amplified by low-fidelity PCR, followed by transformation with linear *RAD50* PCR products into *sae2*Δ cells to replace the corresponding *RAD50* wild-type sequence with the mutagenized DNA fragments. Transformants were then screened for increased viability in the presence of CPT compared with *sae2*Δ cells. This analysis allowed us to identify the *rad50-N18S* mutation, causing the replacement of the Rad50 Arg18 residue with Ser. As shown in Figure 4A, *rad50-N18S* partially suppressed the sensitivity of *sae2*Δ cells not only to CPT but also to MMS.

The *rad50-N18S* and *rif2*Δ alleles suppressed the DNA damage sensitivity of *sae2*Δ cells by altering the same pathway. In support, *sae2*Δ *rif2*Δ *rad50-N18S* triple-mutant cells were resistant to DNA-damaging agents as *sae2*Δ *rif2*Δ double-mutant cells (Figure 4A). The pathway affected by the *rif2*Δ and *rad50-N18S* alleles is different from that altered by the Ku complex, whose removal has been shown to suppress the CPT sensitivity of *sae2*Δ cells by increasing Exo1-mediated resection (Shim et al., 2010; Mimitou and Symington, 2010; Langerak et al., 2011). The *sae2*Δ *ku70*Δ *rad50-N18S* triple-mutant cells were considerably more resistant to DNA-damaging agents than both *sae2*Δ *ku70*Δ and *sae2*Δ *rad50-N18S* double-mutant cells (Figure 4B).

Because the lack of Rif2 decreases the hyperactivation of the checkpoint in *sae2*Δ cells, we investigated the effect of *rad50-N18S* on Rad53 phosphorylation and MRX association at DSBs. Similar to *RIF2* deletion, expression of the *rad50-N18S* allele in *sae2*Δ cells decreased both HO-induced checkpoint activation (Figures 2A and 2B) and Mre11 persistence at the HO-induced DSB (Figure 4C). The decreased Mre11 association at DSBs in *rad50-N18S sae2*Δ cells does not result from reduced DNA binding activity of $MR^{N18S}X$, because the amount of Mre11 bound at DSBs in *rad50-N18S* cells was similar to that of wild-type cells (Figure 4C). The finding that the lack of *RIF2* did not further decrease the amount of Mre11 bound at DSBs in *rad50-N18S sae2*Δ cells (Figure 4C) indicates that the lack of Rif2 and the presence of Rad50-N18S destabilize Mre11 association to DSBs in *sae2*Δ cells by altering the same pathway.

Rif2 inhibits MR endonuclease activity, and MR^{N18S} is refractory to Rif2-mediated inhibition

To gain insight into the function of Rif2 in downregulating MRX association at DSBs in *sae2*Δ cells, we expressed and purified recombinant full-length Rif2 and tested its effect on MR ATPase (Figure 5A) and nuclease activities (Figure 5B). Xrs2 is largely dispensable for the endonuclease activities of the MRX complex (Oh et al., 2016). As previously observed (Cassani et al., 2016; Hailemariam et al., 2019), Rif2 stimulated the ATPase activity of MR (Figure 5A). Interestingly, Rif2 strongly inhibited the endonuclease activity of MR in conjunction with pSae2 (Figures 5C and 5D), whereas it had no effect on its exonuclease activity (Figures 5C and 5E). Because ATPase hydrolysis by Rad50 is required for Sae2 to stimulate Mre11 endonuclease activity (Cannavo and Cejka, 2014; Cannavo et al., 2018), this finding suggests that Rif2-mediated stimulation of ATP hydrolysis by Rad50 leads to a post-hydrolysis MR conformation that is not competent for DNA cleavage.

Because *rad50-N18S* mimics *RIF2* deletion with respect to *sae2*Δ checkpoint inhibition and MRX destabilization at DSBs, we purified MR^{N18S} (Figure 5F) and tested whether the ATPase and nuclease activities of MR^{N18S} are still sensitive to inhibition by Rif2. We observed two main differences compared with wild-type MR. First, Sae2 stimulated the endonuclease activity of MR^{N18S}, although less efficiently than wild-type MR (Figures 5D and 5G). Second, Rif2 was less efficient in inhibiting MR^{N18S} endonuclease activity than inhibiting wild-type MR activity (Figures 5D and 5G), whereas it stimulated the ATPase activity of both MR and MR^{N18S} with similar efficiency (Figure 5A). This finding suggests that Rad50-N18S partially escapes the inhibitory function exerted by Rif2 on Mre11 endonuclease activity.

Rif2 binds a Rad50 surface that is essential for Sae2 to stimulate Mre11 nuclease activity

Because Rif2 is known to interact with Rad50 (Hailemariam et al., 2019), we tested whether the *rad50-N18S* mutation impairs

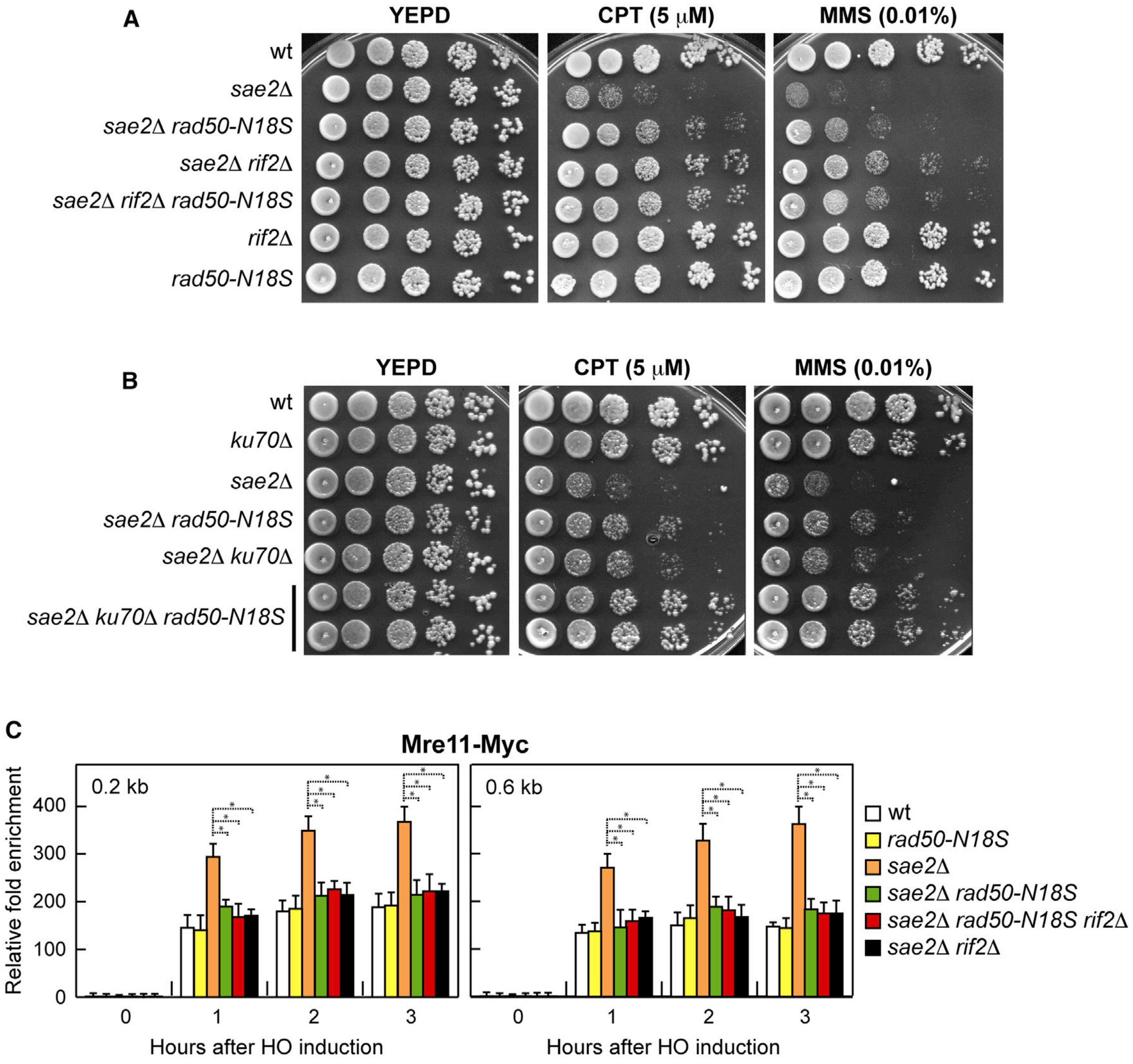

Figure 4. The *rad50-N18S* mutation suppresses the DNA damage sensitivity of *sae2Δ* cells and decreases MRX abundance at DSBs

(A and B) Exponentially growing cultures were serially diluted (1:10), and each dilution was spotted out onto YEPD plates with or without CPT or MMS.

(C) ChIP and qPCR. Exponentially growing YEPR cell cultures were transferred to YEPRG to induce HO expression, followed by ChIP analysis of the recruitment of Mre11-Myc at the indicated distance from the HO-cut. In all diagrams, ChIP signals were normalized for each time point to the corresponding input signal. The mean values of three independent experiments are represented with error bars denoting SD. *$p < 0.005$ (Student's t test).

Rad50-Rif2 interaction. In pull-down assays, Rif2 was able to interact with MR, and the N18S mutation reduced this interaction (Figure 6A), thus explaining the partial escaping of Rif2-mediated inhibition of MR^{N18S} endonuclease activity.

In the ATP-bound state (resting state), Mre11 binding to dsDNA is blocked by Rad50 (Lammens et al., 2011; Williams et al., 2011; Lim et al., 2011; Möckel et al., 2012; Deshpande et al., 2014; Liu et al., 2016; Seifert et al., 2016). A recent cryo-EM of the *E. coli* MR homolog SbcCD has revealed that upon DNA binding and ATP hydrolysis, the two Rad50 coiled-coil domains zip up and, together with the Rad50 NBDs, form a clamp around dsDNA (Käshammer et al., 2019). This structural change allows the Mre11 dimer to move to the side of Rad50, where it binds a DNA end and assembles a DNA cutting channel for nucleolytic reactions (cutting state). Importantly, formation of the cutting state has been shown to require the phosphodiesterase domain of *E. coli* Mre11 to be locked onto Rad50 NBD by a fastener loop in Mre11, which binds the outer β sheet of Rad50 (Käshammer et al., 2019). This interface is important for the nuclease activity of the complex, because mutants losing the binding between Mre11 fastener and Rad50 NBD reduce Mre11 nuclease activity (Käshammer et al., 2019). Although the fastener loop is not conserved in eukaryotes, comparison of the cutting-state structures of *E. coli* sbcCD with that of *S. cerevisiae* MR generated by homology modeling (Cassani et al., 2018) reveals that the fastener loop of *E. coli* sbcC (Mre11 ortholog) binds to the surface of sbcD (Rad50 ortholog) in the same region where a cluster of

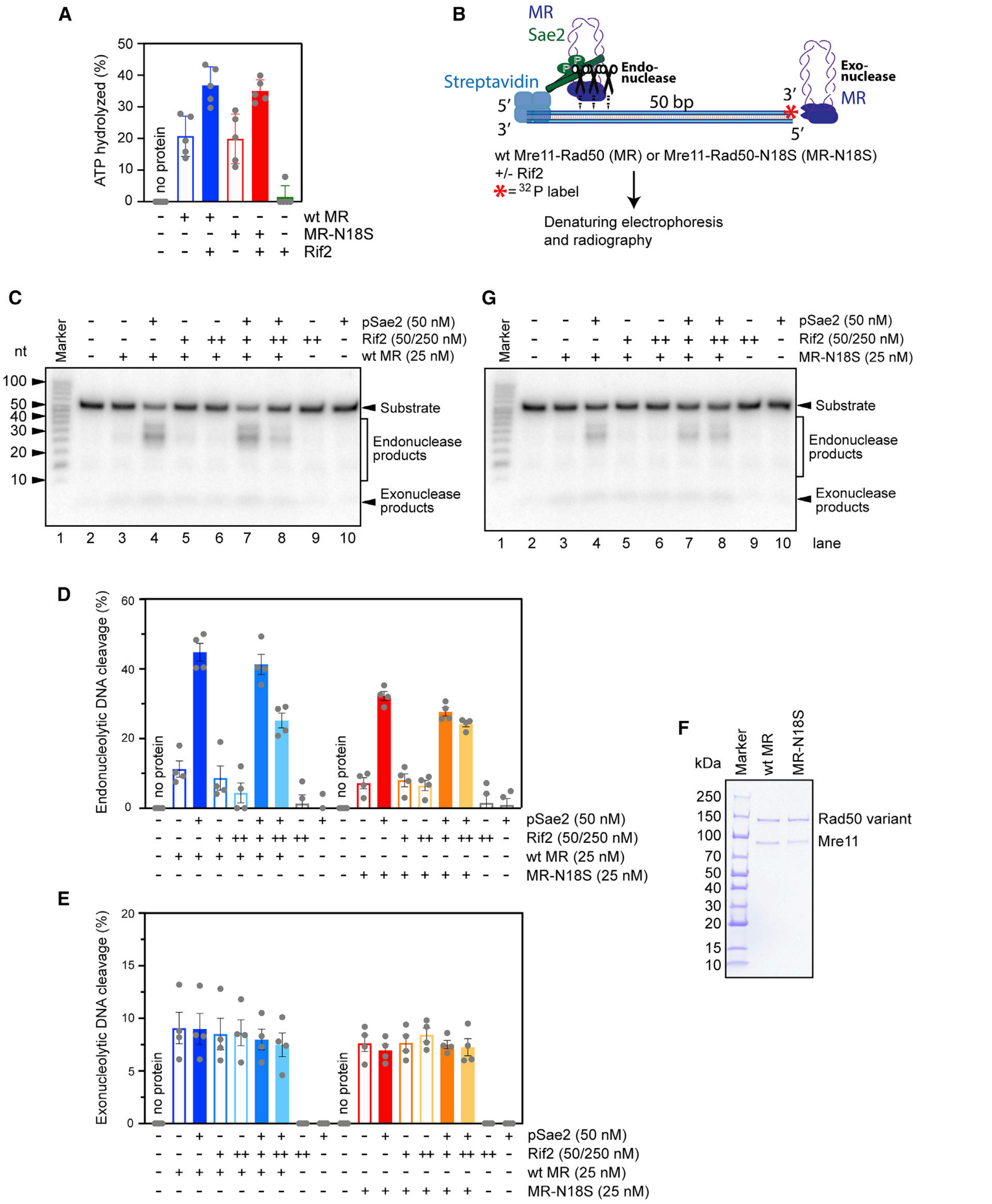

Figure 5. Rif2 inhibits the endonuclease activity of Mre11-Rad50 (MR), but Mre11-Rad50-N18S (MR^{N18S}) escapes this inhibition

(A) Quantitation of ATPase assays was carried out with wild-type MR or MR^{N18S} (both 100 nM) and Rif2 (500 nM), as indicated. Averages are shown from 5 independent experiments; error bars, SEM.

(B) Scheme of the assay used to analyze the effect of Rif2 on the endonuclease activity of MR (which requires phosphorylated Sae2 [pSae2]), and the exonuclease activity of MR. 50-bp-long dsDNA, blocked with streptavidin on one end, was used as substrate.

(legend continued on next page)

Rad50 residues (K6, R20, and K81), corresponding to the *S. cerevisiae rad50-s* mutations, is located (Alani et al., 1990) (Figure 6B). Furthermore, the T188 residue affected by the *mre11-s* mutation in budding yeast (Nairz and Klein, 1997) is localized in the Mre11 α helix facing the same region of the fastener loop (Figure 6B). The *rad50-K81I* mutation, representative of *rad50-s* mutations, is known to impair Mre11 endonuclease activity by disrupting the physical interaction of Sae2 with Rad50 (Cannavo and Cejka, 2014; Cannavo et al., 2018). This finding suggests that instead of the fastener loop, formation of a ternary complex with pSae2 can stabilize the MR NBD interface and therefore the transition to the cutting state.

The N18 residue, which is important for Rad50 to interact with Rif2, is located near the Rad50 K6, R20, and K81 residues altered by the *rad50-s* mutations (Figure 6B), suggesting that Rif2 can also bind to this site. To test this hypothesis, we analyzed whether the Rad50-K81I mutation affects the interaction of Rad50 with Rif2 by coimmunoprecipitation. Because Rif2 binds also Xrs2 and the Rad50-Rif2 interaction occurs in a Xrs2-independent manner (Hirano et al., 2009; Hailemariam et al., 2019), we performed the experiment in *xrs2*Δ cells. When Rif2-Myc was immunoprecipitated with anti-Myc antibodies, a reduced amount of Rad50^{K81I}-hemagglutinin (HA) could be detected in Myc-tagged Rif2 immunoprecipitates compared with wild-type Rad50-HA (Figure 6C). The finding that the K81I mutation weakens the interaction of Rad50 not only with Sae2 but also with Rif2 suggests that Rif2 can inhibit Mre11 endonuclease by binding to this site and interfering with the adoption of the cutting state.

DISCUSSION

pSae2 interacts with Rad50 and stimulates Mre11 endonucleolytic activity through an unknown mechanism (Cannavo and Cejka, 2014). Although Sae2 does not affect the overall ATP hydrolysis rate by Rad50, Sae2-mediated stimulation of MRX endonuclease requires ATP hydrolysis by Rad50, suggesting that Sae2 acts on Rad50 to stimulate Mre11 nuclease activity (Cannavo et al., 2019). The nuclease activity of *E. coli* Mre11 requires the fastener loop, which stabilizes the MR DNA cutting configuration by locking the phosphodiesterase domain of Mre11 onto Rad50 NBD (Käshammer et al., 2019). Although the fastener loop is not conserved in eukaryotes, comparison of bacterial and eukaryotic MR cutting states reveals that the fastener loop of *E. coli* Mre11 binds to the Rad50 residues corresponding to *rad50-s* mutations in yeast Rad50. Because the *rad50-s* mutations abolish Mre11-catalyzed end clipping by abrogating the physical interaction of Rad50 with Sae2 (Alani et al., 1990; Cannavo et al., 2018), we propose that instead of the fastener loop, Sae2 binding to the Rad50-Mre11 interface stabilizes a post-hydrolysis MR cutting conformation, thus explaining the requirement of Sae2 to stimulate Mre11 endonuclease activity (Figure 7).

We show that Mre11 endonuclease activity is inhibited by Rif2 either in the presence or in the absence of Sae2. MRX endonuclease requires ATP hydrolysis by Rad50 (Cannavo et al., 2019). Paradoxically, Rif2 has been shown to stimulate ATP hydrolysis by Rad50 (Cassani et al., 2016; Hailemariam et al., 2019), raising the possibility that this stimulation results in a MR ADP-bound conformation that is not competent to cleave DNA. The Rad50 N18S mutation escapes the Rif2-mediated negative regulation of Mre11 nuclease by weakening Rad50-Rif2 interaction. This mutation, which also renders MR less sensitive to Sae2 stimulation, is located near the Rad50 residues altered by the *rad50-s* mutations (Alani et al., 1990; Cannavo et al., 2018), suggesting that the Rif2 and Sae2 binding sites on Rad50 can partially overlap. In accord with this hypothesis, the *rad50-K81I* mutation, phenocopying the *rad50-s* mutations, impairs not only Sae2-Rad50 interaction but also Rad50-Rif2 interaction. These findings suggest that Rif2 inhibits MR nuclease by antagonizing Sae2 binding to Rad50 and therefore by counteracting the adoption of the endonuclease active cutting state. This Rif2 function can explain why stimulation of Rad50 ATPase activity by Rif2 results into a post-hydrolysis MR conformation that is refractory to Sae2 stimulation of Mre11 nuclease.

Although MR^{N18S} partially escapes the Rif2-mediated inhibition of Mre11 endonuclease activity, Rad50-N18S ATPase activity is still sensitive to Rif2 stimulation. This finding suggests that the residual interaction between Rif2 and Rad50-N18S is sufficient for Rif2 to stimulate ATP hydrolysis by Rad50, but not for counteracting Sae2 binding to Rad50 and therefore for destabilizing the post-hydrolysis MR cutting state.

The lack of either Sae2 or Mre11 nuclease activity increases MRX and Tel1 retention at DSBs, which leads to persistent Rad53 activation that decreases DNA damage resistance (Usui et al., 2001; Clerici et al., 2006; Yu et al., 2018). We found that Rif2 is responsible for the increased MRX and Tel1 association at DSBs in both *sae2*Δ and *mre11-nd* mutant cells and that the *rad50-N18S* mutation escapes the Rif2-mediated increase of MRX retention at DSBs. Rif2 stabilizes MRX and Tel1 association at DSBs only when MRX is nuclease defective. Thus, we propose that failure to stabilize the MRX cutting state, because either Sae2 is absent or Mre11 is catalytically inactive, favors the return to a post-hydrolysis MR conformation that is bound by Rif2 at the MR interface. Rif2 binding stabilizes this ADP-bound MR conformation that is not proficient for DNA cleavage but is still able to bind the DSB ends (Figure 7). Because MRX should move on dsDNA after the cleavage reaction, the stabilization of a catalytically inactive Mre11 by Rif2 can freeze MRX on DNA and generate higher-order oligomeric assemblies that lead to an enhanced association of the complex at DSBs. The increased

(C) Representative nuclease assays with MR, pSae2, and Rif2, as indicated.
(D) Quantitation of endonuclease activity from experiments such as shown in (C) and (G). Averages are shown from 4 independent experiments; error bars, SEM.
(E) Quantitation of exonuclease activity from experiments such as shown in (C) and (G). Averages are shown from 4 independent experiments; error bars, SEM.
(F) MR and MR^{N18S} used in this study.
(G) Representative assays as in (C) but with MR^{N18S}.

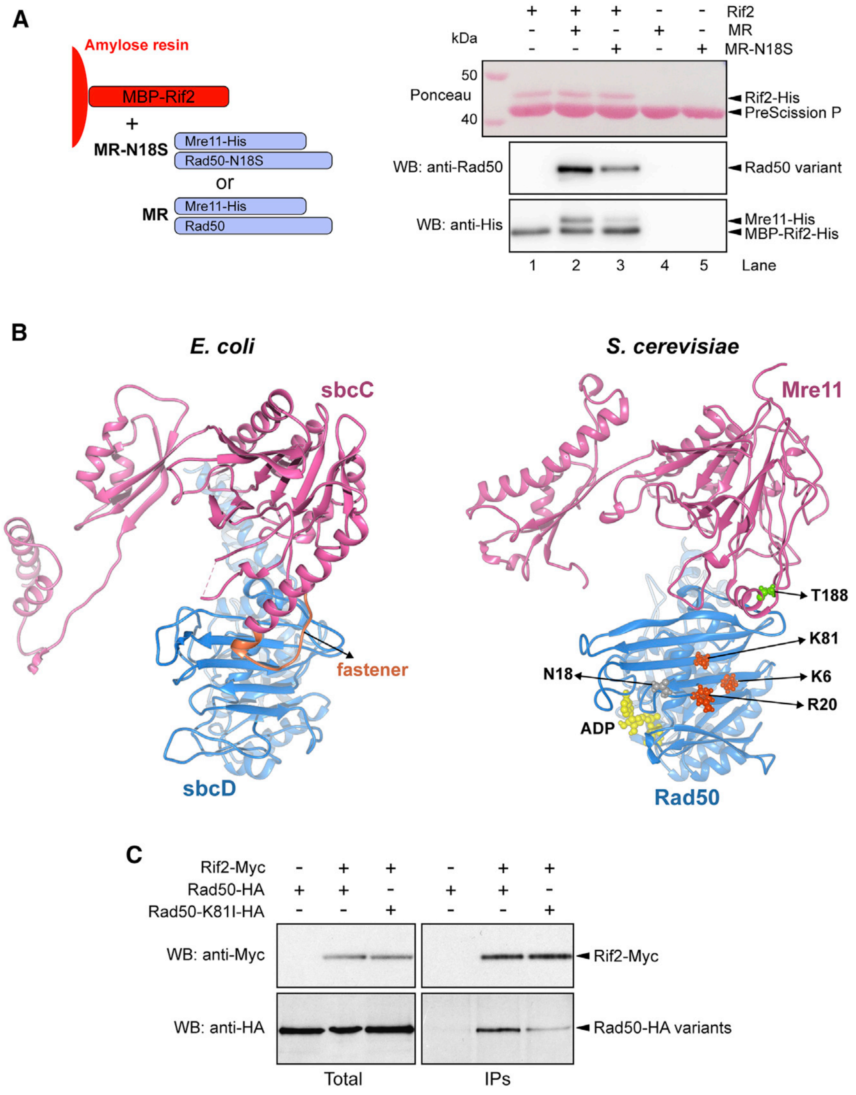

Figure 6. The N18S mutation weakens the Rad50-Rif2 interaction and is located close to the *rad50-s* mutations

(A) Recombinant MBP-Rif2 was immobilized on amylose resin (lanes 1 and 2). Subsequently, MR or MR^{N18S} was added, as indicated. The resin with bound proteins was washed and eluted with maltose, and the MBP tag on Rif2 was cleaved with PreScission protease. The proteins in the eluate were analyzed by Ponceau (Rif2) and western blotting (anti-Rad50 and anti-His to detect Mre11-His).

(B) Comparison of *E. coli* sbcC (Mre11 ortholog, pink) and sbcD (Rad50 ortholog, blue) in the cutting state (PDB: 6S85) and *S. cerevisiae* Mre11 and Rad50 models, obtained by homology modeling, which are positioned by structural alignment to mimic the same cutting-state conformation. The Rad50 residues (K6, R20, and K81) involved in Sae2 interaction and affected by the *rad50-s* mutations are shown as orange spheres. The Mre11 residue (T188) affected by the *mre11-s* mutation is shown as green spheres. The Rad50 residue N18 is drawn in gray spheres, whereas the ADP nucleotide is in yellow.

(C) Protein extracts from exponentially growing *xrs2*Δ cells were analyzed by western blotting with anti-HA and anti-Myc antibodies either directly (total) or after immunoprecipitation (IP) with anti-Myc antibody.

MRX retention at DSBs occurs only when Mre11 is nuclease defective, possibly because the amount of Rif2 at DSBs is not enough to counteract Sae2-mediated stabilization of the MRX cutting state.

Which are the biological consequences of Rif2-mediated MRX regulation when Sae2 is present? DSBs are more avidly bound by Sae2 than by Rif2, whereas the opposite occurs at telomeres, where Rif2, together with Rif1, gives rise to a higher-order architecture that interlinks Rap1 units (Shi et al., 2013). The lack of Rif2 by itself was shown to increase NHEJ at both DSBs and telomeres (Marcand et al., 2008; Cassani et al., 2016). Because NHEJ has been shown to rely on the MRX ATP-bound state (Deshpande et al., 2014; Cassani et al., 2018), which generates a groove that can host dsDNA (Lammens et al., 2011; Rojowska et al., 2014; Liu et al., 2016; Seifert et al., 2016), Rif2 can inhibit NHEJ by discharging the MRX ATP-bound state though stimulation of ATP hydrolysis by Rad50. This Rif2-mediated discharge of the MRX ATP-bound state can also explain why the lack of Rif2 by itself leads to an increased MRX and Tel1 association at both DSBs and telomeres (Hirano et al., 2009; Cassani et al., 2016).

By contrast, the lack of Rif2 does not enhance hairpin cleavage or resection of mitotic and meiotic DSBs. Because these MRX functions strictly depend on Sae2, whose association to DSBs is higher than that of Rif2, the amount of Rif2 bound at DSBs cannot be enough to antagonize Sae2 binding to Rad50. The low amount of Rif2 bound at DSBs compared with Sae2 ensures that cells possess a sufficient amount of ATP-bound MRX at DSBs that can be converted by Sae2 to an endonuclease active state.

By contrast, Rif2 inhibits MRX-mediated resection at telomeres (Bonetti et al., 2010; Hirano et al., 2009; McGee et al., 2010; Ribeyre and Shore, 2012), where the high Rif2 level can destabilize the MRX cutting state even in the presence of Sae2. Thus, although Rif2 at DSBs has an inhibitory role on NHEJ by discharging the MRX ATP-bound state, contributing to regulate the choice between NHEJ and HR, at telomeres Rif2 ensures protection of telomeric DNA ends from MRX-Sae2-mediated nucleolytic degradation. The finding that Rif2 directly binds DNA *in vitro* raises the possibility that Rif2-mediated inhibition of Mre11 endonuclease activity can also be important to protect internal DNA regions from unscheduled endonucleolytic scission by MRX-Sae2. Because the MRX complex is evolutionarily conserved, it will be interesting to investigate whether a similar inhibitory mechanism occurs on human MRN.

CellPress
OPEN ACCESS

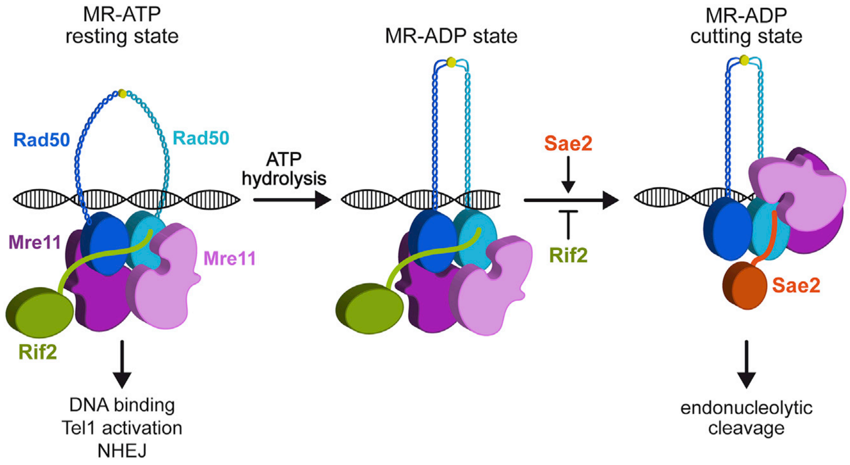

Figure 7. Model for Sae2 and Rif2 regulation of MRX activity at DNA ends

The MRX ATP-bound state (resting state) can bind DNA and promotes NHEJ and Tel1 activation. In this state, Mre11 embraces Rad50-ATP dimer, resulting in Mre11 being inaccessible to dsDNA and therefore not competent to cleave DNA. After DSB recognition and ATP hydrolysis, Rad50 adopts a conformation that induces a rotation of the coiled-coils arms, which clamp upon DNA binding. The Mre11 dimer is able to move from the Rad50 dimer and reaches the DNA end. Sae2 binding on the Rad50-Mre11 interface stabilizes the resulting conformation (cutting state) that is proficient to cleave DNA. Rif2 binding near the Sae2-interacting site on Rad50 inhibits the conversion to the endonuclease active cutting state and stabilizes a MRX ADP-bound state that is not competent for DNA cleavage but can bind DNA.

STAR★METHODS

Detailed methods are provided in the online version of this paper and include the following:

- KEY RESOURCES TABLE
- RESOURCE AVAILABILITY
  - Lead contact
  - Materials availability
  - Data and code availability
- EXPERIMENTAL MODEL AND SUBJECT DETAILS
- METHOD DETAILS
  - Yeast media
  - Search for rad50 mutants suppressing the sensitivity to CPT of sae2Δ cells
  - Spot assays
  - Western blotting and immunoprecipitation
  - Chromatin Immunoprecipitation and qPCR
  - Preparation of recombinant proteins
  - ATPase assays
  - Nuclease assays
  - Protein interaction assays
- QUANTIFICATION AND STATISTICAL ANALYSIS

SUPPLEMENTAL INFORMATION

Supplemental information can be found online at https://doi.org/10.1016/j.celrep.2021.108906.

ACKNOWLEDGMENTS

We thank J. Haber and M. Resnick for yeast strains, A. Bianchi for Rif2 expression plasmid, G. Monticelli for preliminary data, and M. Clerici and G. Lucchini for critical reading of the manuscript. This work was supported by Fondazione AIRC under IG 2017-ID. 19783 and Progetti di Ricerca di Interesse Nazionale (PRIN) 2017 to M.P.L., the Swiss National Science Foundation (grant 31003A_175444) to P.C., and the European Research Council (grant 681630) to P.C.

AUTHOR CONTRIBUTIONS

A.M. and M.P.L. designed the genetic experiments, and E.C. and P.C. designed the biochemical experiments. A.M., E.G., and C.C. performed the genetic experiments, and E.C. performed the biochemical experiments. G.R. prepared the recombinant proteins. A.M., R.T., E.C., P.C., and M.P.L. analyzed the data. M.P.L. wrote the paper.

DECLARATION OF INTERESTS

The authors declare no competing interests.

Received: October 26, 2020
Revised: January 29, 2021
Accepted: March 4, 2021
Published: March 30, 2021

REFERENCES

Alani, E., Padmore, R., and Kleckner, N. (1990). Analysis of wild-type and *rad50* mutants of yeast suggests an intimate relationship between meiotic chromosome synapsis and recombination. Cell *61*, 419–436.

Anand, R., Ranjha, L., Cannavo, E., and Cejka, P. (2016). Phosphorylated CtIP functions as a co-factor of the MRE11-RAD50-NBS1 endonuclease in DNA end resection. Mol. Cell *64*, 940–950.

Bergerat, A., de Massy, B., Gadelle, D., Varoutas, P.C., Nicolas, A., and Forterre, P. (1997). An atypical topoisomerase II from Archaea with implications for meiotic recombination. Nature *386*, 414–417.

Bonetti, D., Clerici, M., Anbalagan, S., Martina, M., Lucchini, G., and Longhese, M.P. (2010). Shelterin-like proteins and Yku inhibit nucleolytic processing of *Saccharomyces cerevisiae* telomeres. PLoS Genet. *6*, e1000966.

Cannavo, E., and Cejka, P. (2014). Sae2 promotes dsDNA endonuclease activity within Mre11-Rad50-Xrs2 to resect DNA breaks. Nature *514*, 122–125.

Cannavo, E., Johnson, D., Andres, S.N., Kissling, V.M., Reinert, J.K., Garcia, V., Erie, D.A., Hess, D., Thomä, N.H., Enchev, R.I., et al. (2018). Regulatory control of DNA end resection by Sae2 phosphorylation. Nat. Commun. *9*, 4016.

Cannavo, E., Reginato, G., and Cejka, P. (2019). Stepwise 5′ DNA end-specific resection of DNA breaks by the Mre11-Rad50-Xrs2 and Sae2 nuclease ensemble. Proc. Natl. Acad. Sci. USA *116*, 5505–5513.

Cassani, C., Gobbini, E., Wang, W., Niu, H., Clerici, M., Sung, P., and Longhese, M.P. (2016). Tel1 and Rif2 regulate MRX functions in end-tethering and repair of DNA double-strand breaks. PLoS Biol. *14*, e1002387.

Cassani, C., Gobbini, E., Vertemara, J., Wang, W., Marsella, A., Sung, P., Tisi, R., Zampella, G., and Longhese, M.P. (2018). Structurally distinct Mre11 domains mediate MRX functions in resection, end-tethering and DNA damage resistance. Nucleic Acids Res. *46*, 2990–3008.

Cejka, P., Cannavo, E., Polaczek, P., Masuda-Sasa, T., Pokharel, S., Campbell, J.L., and Kowalczykowski, S.C. (2010). DNA end resection by Dna2-Sgs1-RPA

and its stimulation by Top3-Rmi1 and Mre11-Rad50-Xrs2. Nature *467*, 112–116.

Chen, H., Donnianni, R.A., Handa, N., Deng, S.K., Oh, J., Timashev, L.A., Kowalczykowski, S.C., and Symington, L.S. (2015). Sae2 promotes DNA damage resistance by removing the Mre11-Rad50-Xrs2 complex from DNA and attenuating Rad53 signaling. Proc. Natl. Acad. Sci. USA *112*, E1880–E1887.

Clerici, M., Mantiero, D., Lucchini, G., and Longhese, M.P. (2006). The *Saccharomyces cerevisiae* Sae2 protein negatively regulates DNA damage checkpoint signalling. EMBO Rep. *7*, 212–218.

Colombo, C.V., Menin, L., Ranieri, R., Bonetti, D., Clerici, M., and Longhese, M.P. (2019). Uncoupling Sae2 functions in downregulation of Tel1 and Rad53 signaling activities. Genetics *211*, 515–530.

Deshpande, R.A., Williams, G.J., Limbo, O., Williams, R.S., Kuhnlein, J., Lee, J.H., Classen, S., Guenther, G., Russell, P., Tainer, J.A., and Paull, T.T. (2014). ATP-driven Rad50 conformations regulate DNA tethering, end resection, and ATM checkpoint signaling. EMBO J. *33*, 482–500.

Ferrari, M., Dibitetto, D., De Gregorio, G., Eapen, V.V., Rawal, C.C., Lazzaro, F., Tsabar, M., Marini, F., Haber, J.E., and Pellicioli, A. (2015). Functional interplay between the 53BP1-ortholog Rad9 and the Mre11 complex regulates resection, end-tethering and repair of a double-strand break. PLoS Genet. *11*, e1004928.

Garcia, V., Phelps, S.E.L., Gray, S., and Neale, M.J. (2011). Bidirectional resection of DNA double-strand breaks by Mre11 and Exo1. Nature *479*, 241–244.

Gilbert, C.S., Green, C.M., and Lowndes, N.F. (2001). Budding yeast Rad9 is an ATP-dependent Rad53 activating machine. Mol. Cell *8*, 129–136.

Gobbini, E., Villa, M., Gnugnoli, M., Menin, L., Clerici, M., and Longhese, M.P. (2015). Sae2 function at DNA double-strand breaks is bypassed by dampening Tel1 or Rad53 activity. PLoS Genet. *11*, e1005685.

Hailemariam, S., De Bona, P., Galletto, R., Hohl, M., Petrini, J.H., and Burgers, P.M. (2019). The telomere-binding protein Rif2 and ATP-bound Rad50 have opposing roles in the activation of yeast Tel1^{ATM} kinase. J. Biol. Chem. *294*, 18846–18852.

Hirano, Y., Fukunaga, K., and Sugimoto, K. (2009). Rif1 and rif2 inhibit localization of tel1 to DNA ends. Mol. Cell *33*, 312–322.

Hopfner, K.P., Karcher, A., Shin, D., Fairley, C., Tainer, J.A., and Carney, J.P. (2000). Mre11 and Rad50 from *Pyrococcus furiosus*: cloning and biochemical characterization reveal an evolutionarily conserved multiprotein machine. J. Bacteriol. *182*, 6036–6041.

Hopfner, K.P., Karcher, A., Craig, L., Woo, T.T., Carney, J.P., and Tainer, J.A. (2001). Structural biochemistry and interaction architecture of the DNA double-strand break repair Mre11 nuclease and Rad50-ATPase. Cell *105*, 473–485.

Hopfner, K.P., Craig, L., Moncalian, G., Zinkel, R.A., Usui, T., Owen, B.A., Karcher, A., Henderson, B., Bodmer, J.L., McMurray, C.T., et al. (2002). The Rad50 zinc-hook is a structure joining Mre11 complexes in DNA recombination and repair. Nature *418*, 562–566.

Kaizer, H., Connelly, C.J., Bettridge, K., Viggiani, C., and Greider, C.W. (2015). Regulation of telomere length requires a conserved N-terminal domain of Rif2 in *Saccharomyces cerevisiae*. Genetics *201*, 573–586.

Käshammer, L., Saathoff, J.H., Lammens, K., Gut, F., Bartho, J., Alt, A., Kessler, B., and Hopfner, K.P. (2019). Mechanism of DNA end sensing and processing by the Mre11-Rad50 complex. Mol. Cell *76*, 382–394.e6.

Keeney, S., Giroux, C.N., and Kleckner, N. (1997). Meiosis-specific DNA double-strand breaks are catalyzed by Spo11, a member of a widely conserved protein family. Cell *88*, 375–384.

Lammens, K., Bemeleit, D.J., Möckel, C., Clausing, E., Schele, A., Hartung, S., Schiller, C.B., Lucas, M., Angermüller, C., Söding, J., et al. (2011). The Mre11:Rad50 structure shows an ATP-dependent molecular clamp in DNA double-strand break repair. Cell *145*, 54–66.

Langerak, P., Mejia-Ramirez, E., Limbo, O., and Russell, P. (2011). Release of Ku and MRN from DNA ends by Mre11 nuclease activity and Ctp1 is required for homologous recombination repair of double-strand breaks. PLoS Genet. *7*, e1002271.

Lee, S.E., Moore, J.K., Holmes, A., Umezu, K., Kolodner, R.D., and Haber, J.E. (1998). *Saccharomyces* Ku70, mre11/rad50 and RPA proteins regulate adaptation to G2/M arrest after DNA damage. Cell *94*, 399–409.

Levy, D.L., and Blackburn, E.H. (2004). Counting of Rif1p and Rif2p on *Saccharomyces cerevisiae* telomeres regulates telomere length. Mol. Cell. Biol. *24*, 10857–10867.

Lim, H.S., Kim, J.S., Park, Y.B., Gwon, G.H., and Cho, Y. (2011). Crystal structure of the Mre11-Rad50-ATPγS complex: understanding the interplay between Mre11 and Rad50. Genes Dev. *25*, 1091–1104.

Lisby, M., Barlow, J.H., Burgess, R.C., and Rothstein, R. (2004). Choreography of the DNA damage response: spatiotemporal relationships among checkpoint and repair proteins. Cell *118*, 699–713.

Liu, Y., Sung, S., Kim, Y., Li, F., Gwon, G., Jo, A., Kim, A.K., Kim, T., Song, O.K., Lee, S.E., and Cho, Y. (2016). ATP-dependent DNA binding, unwinding, and resection by the Mre11/Rad50 complex. EMBO J. *35*, 743–758.

Lobachev, K.S., Gordenin, D.A., and Resnick, M.A. (2002). The Mre11 complex is required for repair of hairpin-capped double-strand breaks and prevention of chromosome rearrangements. Cell *108*, 183–193.

Marcand, S., Pardo, B., Gratias, A., Cahun, S., and Callebaut, I. (2008). Multiple pathways inhibit NHEJ at telomeres. Genes Dev. *22*, 1153–1158.

Martina, M., Clerici, M., Baldo, V., Bonetti, D., Lucchini, G., and Longhese, M.P. (2012). A balance between Tel1 and Rif2 activities regulates nucleolytic processing and elongation at telomeres. Mol. Cell. Biol. *32*, 1604–1617.

McGee, J.S., Phillips, J.A., Chan, A., Sabourin, M., Paeschke, K., and Zakian, V.A. (2010). Reduced Rif2 and lack of Mec1 target short telomeres for elongation rather than double-strand break repair. Nat. Struct. Mol. Biol. *17*, 1438–1445.

McKee, A.H., and Kleckner, N. (1997). A general method for identifying recessive diploid-specific mutations in *Saccharomyces cerevisiae*, its application to the isolation of mutants blocked at intermediate stages of meiotic prophase and characterization of a new gene *SAE2*. Genetics *146*, 797–816.

Mehta, A., and Haber, J.E. (2014). Sources of DNA double-strand breaks and models of recombinational DNA repair. Cold Spring Harb. Perspect. Biol. *6*, a016428.

Mimitou, E.P., and Symington, L.S. (2008). Sae2, Exo1 and Sgs1 collaborate in DNA double-strand break processing. Nature *455*, 770–774.

Mimitou, E.P., and Symington, L.S. (2010). Ku prevents Exo1 and Sgs1-dependent resection of DNA ends in the absence of a functional MRX complex or Sae2. EMBO J. *29*, 3358–3369.

Möckel, C., Lammens, K., Schele, A., and Hopfner, K.P. (2012). ATP driven structural changes of the bacterial Mre11:Rad50 catalytic head complex. Nucleic Acids Res. *40*, 914–927.

Moreau, S., Ferguson, J.R., and Symington, L.S. (1999). The nuclease activity of Mre11 is required for meiosis but not for mating type switching, end joining, or telomere maintenance. Mol. Cell. Biol. *19*, 556–566.

Moreno-Herrero, F., de Jager, M., Dekker, N.H., Kanaar, R., Wyman, C., and Dekker, C. (2005). Mesoscale conformational changes in the DNA-repair complex Rad50/Mre11/Nbs1 upon binding DNA. Nature *437*, 440–443.

Nairz, K., and Klein, F. (1997). *mre11S*—a yeast mutation that blocks double-strand-break processing and permits nonhomologous synapsis in meiosis. Genes Dev. *11*, 2272–2290.

Neale, M.J., Pan, J., and Keeney, S. (2005). Endonucleolytic processing of covalent protein-linked DNA double-strand breaks. Nature *436*, 1053–1057.

Niu, H., Chung, W.H., Zhu, Z., Kwon, Y., Zhao, W., Chi, P., Prakash, R., Seong, C., Liu, D., Lu, L., et al. (2010). Mechanism of the ATP-dependent DNA end-resection machinery from *Saccharomyces cerevisiae*. Nature *467*, 108–111.

Oh, J., Al-Zain, A., Cannavo, E., Cejka, P., and Symington, L.S. (2016). Xrs2 dependent and independent functions of the Mre11-Rad50 complex. Mol. Cell *64*, 405–415.

Park, Y.B., Hohl, M., Padjasek, M., Jeong, E., Jin, K.S., Krężel, A., Petrini, J.H., and Cho, Y. (2017). Eukaryotic Rad50 functions as a rod-shaped dimer. Nat. Struct. Mol. Biol. *24*, 248–257.

CellPress
OPEN ACCESS

Prinz, S., Amon, A., and Klein, F. (1997). Isolation of *COM1*, a new gene required to complete meiotic double-strand break-induced recombination in *Saccharomyces cerevisiae*. Genetics *146*, 781–795.

Puddu, F., Oelschlaegel, T., Guerini, I., Geisler, N.J., Niu, H., Herzog, M., Salguero, I., Ochoa-Montaño, B., Viré, E., Sung, P., et al. (2015). Synthetic viability genomic screening defines Sae2 function in DNA repair. EMBO J. *34*, 1509–1522.

Ranjha, L., Howard, S.M., and Cejka, P. (2018). Main steps in DNA double-strand break repair: an introduction to homologous recombination and related processes. Chromosoma *127*, 187–214.

Reginato, G., and Cejka, P. (2020). The MRE11 complex: A versatile toolkit for the repair of broken DNA. DNA Repair (Amst.) *91–92*, 102869.

Reginato, G., Cannavo, E., and Cejka, P. (2017). Physiological protein blocks direct the Mre11-Rad50-Xrs2 and Sae2 nuclease complex to initiate DNA end resection. Genes Dev. *31*, 2325–2330.

Ribeyre, C., and Shore, D. (2012). Anticheckpoint pathways at telomeres in yeast. Nat. Struct. Mol. Biol. *19*, 307–313.

Rojowska, A., Lammens, K., Seifert, F.U., Direnberger, C., Feldmann, H., and Hopfner, K.P. (2014). Structure of the Rad50 DNA double-strand break repair protein in complex with DNA. EMBO J. *33*, 2847–2859.

Schwartz, M.F., Duong, J.K., Sun, Z., Morrow, J.S., Pradhan, D., and Stern, D.F. (2002). Rad9 phosphorylation sites couple Rad53 to the *Saccharomyces cerevisiae* DNA damage checkpoint. Mol. Cell *9*, 1055–1065.

Seifert, F.U., Lammens, K., Stoehr, G., Kessler, B., and Hopfner, K.P. (2016). Structural mechanism of ATP-dependent DNA binding and DNA end bridging by eukaryotic Rad50. EMBO J. *35*, 759–772.

Shi, T., Bunker, R.D., Mattarocci, S., Ribeyre, C., Faty, M., Gut, H., Scrima, A., Rass, U., Rubin, S.M., Shore, D., and Thomä, N.H. (2013). Rif1 and Rif2 shape telomere function and architecture through multivalent Rap1 interactions. Cell *153*, 1340–1353.

Shibata, A., Moiani, D., Arvai, A.S., Perry, J., Harding, S.M., Genois, M.M., Maity, R., van Rossum-Fikkert, S., Kertokalio, A., Romoli, F., et al. (2014). DNA double-strand break repair pathway choice is directed by distinct MRE11 nuclease activities. Mol. Cell *53*, 7–18.

Shim, E.Y., Chung, W.H., Nicolette, M.L., Zhang, Y., Davis, M., Zhu, Z., Paull, T.T., Ira, G., and Lee, S.E. (2010). *Saccharomyces cerevisiae* Mre11/Rad50/Xrs2 and Ku proteins regulate association of Exo1 and Dna2 with DNA breaks. EMBO J. *29*, 3370–3380.

Sweeney, F.D., Yang, F., Chi, A., Shabanowitz, J., Hunt, D.F., and Durocher, D. (2005). *Saccharomyces cerevisiae* Rad9 acts as a Mec1 adaptor to allow Rad53 activation. Curr. Biol. *15*, 1364–1375.

Syed, A., and Tainer, J.A. (2018). The MRE11-RAD50-NBS1 complex conducts the orchestration of damage signaling and outcomes to stress in DNA replication and repair. Annu. Rev. Biochem. *87*, 263–294.

Tisi, R., Vertemara, J., Zampella, G., and Longhese, M.P. (2020). Functional and structural insights into the MRX/MRN complex, a key player in recognition and repair of DNA double-strand breaks. Comput. Struct. Biotechnol. J. *18*, 1137–1152.

Trujillo, K.M., and Sung, P. (2001). DNA structure-specific nuclease activities in the *Saccharomyces cerevisiae* Rad50*Mre11 complex. J. Biol. Chem. *276*, 35458–35464.

Usui, T., Ohta, T., Oshiumi, H., Tomizawa, J., Ogawa, H., and Ogawa, T. (1998). Complex formation and functional versatility of Mre11 of budding yeast in recombination. Cell *95*, 705–716.

Usui, T., Ogawa, H., and Petrini, J.H. (2001). A DNA damage response pathway controlled by Tel1 and the Mre11 complex. Mol. Cell *7*, 1255–1266.

Villa, M., Cassani, C., Gobbini, E., Bonetti, D., and Longhese, M.P. (2016). Coupling end resection with the checkpoint response at DNA double-strand breaks. Cell. Mol. Life Sci. *73*, 3655–3663.

Wang, W., Daley, J.M., Kwon, Y., Krasner, D.S., and Sung, P. (2017). Plasticity of the Mre11-Rad50-Xrs2-Sae2 nuclease ensemble in the processing of DNA-bound obstacles. Genes Dev. *31*, 2331–2336.

Williams, R.S., Moncalian, G., Williams, J.S., Yamada, Y., Limbo, O., Shin, D.S., Groocock, L.M., Cahill, D., Hitomi, C., Guenther, G., et al. (2008). Mre11 dimers coordinate DNA end bridging and nuclease processing in double-strand-break repair. Cell *135*, 97–109.

Williams, G.J., Williams, R.S., Williams, J.S., Moncalian, G., Arvai, A.S., Limbo, O., Guenther, G., SilDas, S., Hammel, M., Russell, P., and Tainer, J.A. (2011). ABC ATPase signature helices in Rad50 link nucleotide state to Mre11 interface for DNA repair. Nat. Struct. Mol. Biol. *18*, 423–431.

Wotton, D., and Shore, D. (1997). A novel Rap1p-interacting factor, Rif2p, cooperates with Rif1p to regulate telomere length in *Saccharomyces cerevisiae*. Genes Dev. *11*, 748–760.

Yu, T.Y., Kimble, M.T., and Symington, L.S. (2018). Sae2 antagonizes Rad9 accumulation at DNA double-strand breaks to attenuate checkpoint signaling and facilitate end resection. Proc. Natl. Acad. Sci. USA *115*, E11961–E11969.

Zhu, Z., Chung, W.H., Shim, E.Y., Lee, S.E., and Ira, G. (2008). Sgs1 helicase and two nucleases Dna2 and Exo1 resect DNA double-strand break ends. Cell *134*, 981–994.

STAR★METHODS

KEY RESOURCES TABLE

REAGENT or RESOURCE	SOURCE	IDENTIFIER
Antibodies		
Anti-Rad53	Abcam	Ab104232; RRID: AB_2687603
Anti-Myc (9E10)	In house antibody	N/A
Anti-HA (12CA5)	In house antibody	N/A
Anti-Rad50	Thermo Scientific	PA5-32176; RRID: AB_2549649
Anti-His	MBL	D291-3; RRID: 10597733
Bacterial and virus strains		
Subcloning Efficiency DH5alpha Competent Cells	Invitrogen	18265017
MAX Efficiency DH10Bac Competent Cells	GIBCO	10361012
Chemicals, peptides, and recombinant proteins		
SsoFast™ EvaGreen®Supermix, 500 Rxn	Bio-Rad	1725201
Hygromycin B	Roche	10843555001
ClonNAT (nourseothricin)	WERNERBioAgents	96736-11-7
G-418 disulfate	Medchemexpress	HY-17561
(S)-(+)-Camptothecin	Sigma-Aldrich	C9911-1G
Methyl methanesulfonate	Sigma-Aldrich	129925/25G
Trichloroacetic acid	Sigma-Aldrich	91230-1KG
RNase A	Roche	10109169001
Bromophenol Blue sodium salt	Sigma-Aldrich	B6131-25G
Phenylmethanesulfonyl fluoride	Sigma-Aldrich	78830-5G
Sodium chloride	Sigma-Aldrich	31434-1KG-R-D
Hydrochloric acid	Sigma-Aldrich	30721-1L-M
Yeast nitrogen base with amino acids	Sigma-Aldrich	Y1250-250G
Dimethyl sulfoxide	Sigma-Aldrich	D4540-1L
Triton® X-100 for molecular biology	Sigma-Aldrich	T8787-100ML
Sodium deoxycholate	Sigma-Aldrich	30970-100G
Lithium chloride	Sigma-Aldrich	L9650-100G
N,N,N',N'-Tetramethylethylenediamine	Sigma-Aldrich	T9281-50ML
Acrilamide 4X solution	Serva	10677.1
N,N'-Methylene-bisacrylamide 2X	Serva	29197.01
IGEPAL® CA-630	Sigma-Aldrich	I8896-100ML
Ammonium persulfate	Sigma-Aldrich	A3678-25G
DL-Dithiothreitol	Sigma-Aldrich	43819-25G
HEPES	Sigma-Aldrich	H4034-1KG
Ethylenediaminetetraacetic acid $\geq$ 98.0%	Sigma-Aldrich	03620-1KG
Complete Mini	Roche	11836153001
Peroxide solution and Enhancer solution	Genespin	STS-E500
D-Sorbitol	Sigma-Aldrich	S7547-1KG
Ponceau s sodium practical grade	Sigma-Aldrich	P3504-100G
Trizma® base	Sigma-Aldrich	33742-2KG
Sodium dodecyl sulfate	Sigma-Aldrich	L3771-500G
Sodium hydroxyde	Merck Millipore	1064621000
Formaldehyde solution for molecular biology, 36.5–38% in H2O	Sigma-Aldrich	F8775-500ML
Glycine for electrophoresis, $\geq$ 99%	Sigma-Aldrich	G8898-1KG
2-Propanol	Sigma-Aldrich	I9516-500ML

(*Continued on next page*)

Continued

REAGENT or RESOURCE	SOURCE	IDENTIFIER
Ethanol absolute	Sigma-Aldrich	02860-2,5L
Zymolyase 20T	Nacalai Tesque	07663-91
Dynabeads Protein G	Invitrogen	10004D
Agarose LE	Euroclone	EMR920500-500 g
D(+)-Raffinose pentahydrate	Sigma-Aldrich	83400-100G
D(+)-Galactose	Sigma-Aldrich	48260-500G-F
D-(+)-Glucose monohydrate	Sigma-Aldrich	49159-5KG
Yeast Extract Difco	BD	212750
Peptone Difco	BD	211677
Peptone Oxoid	OXOID	LP0037T
Yeast extract Oxoid	OXOID	LP0021T
Agar Bacto Difco	BD	214030
Agarose LE	EuroClone	EMR920500-500 g
SFX-Insect with L-glutamine and sodium bicarbonate	HyClone	HYCLSH30278
*Trans*IT®-Insect Transfection Reagent	Mirus	MIR 6100
Dulbecco's Phosphate Buffered Saline	Sigma-Aldrich	D8537-500ML
Protease Inhibitor Cocktail	Sigma-Aldrich	P8340
Leupeptin	Merck	EI8
Glycerol	PanReac AppliChem	A2926.2500
Potassium phosphate monobasic	Sigma-Aldrich	P9791-500G
Potassium phosphate dibasic	Sigma-Aldrich	P2222-500G
Potassium chloride	Sigma-Aldrich	P9541-1KG
2-Mercaptoethanol	Sigma-Aldrich	M6250-100ML
Ni-NTA Agarose	QIAGEN	30230
Imidazole	Sigma-Aldrich	I202-500G
HiTrap® SP HP	Cytiva	GE29-0513-24
HiTrap® Q HP	Cytiva	GE29-0513-25
EDTA	Sigma-Aldrich	EDS
PMSF	AppliChem	A0999
Nonidet P40 Substitute	Sigma-Aldrich	74385
D-(+)-Maltose monohydrate	Sigma-Aldrich	M9171
Amylose resin	New England Biolabs	E8021L
ATP, [γ-32P]- 3000Ci/mmol 10mCi/ml	Perkin Elmer	BLU502A500UC
BSA	BioConcept AG	B9000S
ATP	Cytiva	GE-27-1006-03
Pyruvate Kinase from rabbit muscle	Sigma-Aldrich	P1506-5K
PEP-Phospho (enol) pyruvic acid	Sigma-Aldrich	P7002
**Critical commercial assays**		
QIAGEN QIAquick PCR Purification kit	QIAGEN	28106
QIAGEN Plasmid Mini Kit	QIAGEN	12125
Bac-to-Bac Baculovirus Expression System	GIBCO	10359016
**Experimental models: Organisms/strains**		
S. cerevisiae, see Table S1	This study	N/A
**Oligonucleotides**		
ARO+: TGAGTCGTTACAAGGTGATGCC	This study	N/A
ARO-: ACCTACAGGAGGACCCGAAA	This study	N/A
DSB 0.2+ TCAGACTCAAGCAAACAATCAA	This study	N/A
DSB 0.2-: CCCGTATAGCCAATTCGTTC	This study	N/A

(*Continued on next page*)

Continued		
REAGENT or RESOURCE	SOURCE	IDENTIFIER
DSB 0.6+: CACCCAAGAAGGCGAATAAG	This study	N/A
DSB 0.6-: CATGCGGTTCACATGACTTT	This study	N/A
PC1253C:AACGTCATAGACGATTACATTGCTAG GACATCTTTGCCCACGTTGACCCA	Cannavo and Cejka, 2014	N/A
PC1253B:TGGGTCAACGTGGGCAAAGATGTCCT AGCAATGTAATCGTCTATGACGTT with 3′terminal biotin	Cannavo and Cejka, 2014	N/A
Software and algorithms		
Bio-Rad CFX Maestro 1.1 Version: 4.1.2433.1219	Bio-Rad	
Other		
White 96-well PCR plates Multiplate™	Bio-Rad	MLL9651
Nitrocellulose blotting membrane, Amersham™ Protran™ 0.45 μm NC	GE Healthcare	10600002
TLC Plastic Sheets	Sigma-Aldrich	Z740237

RESOURCE AVAILABILITY

Lead contact

Further information and requests for resources and reagents should be directed to and will be fulfilled by the lead contact, Maria Pia Longhese (mariapia.longhese@unimib.it).

Materials availability

All unique/stable reagents generated in this study are available from the lead contact without restriction.

Data and code availability

This study did not generate any unique datasets or code.

EXPERIMENTAL MODEL AND SUBJECT DETAILS

Saccharomyces cerevisiae is the experimental model used in this study. Strain genotypes are listed in Table S1. Strain JKM139, used to detect DSB resection, was kindly provided by J. Haber (Brandeis University, Waltham, USA). Strain HS21, used to detect hairpin cleavage, was kindly provided by M. A. Resnick (NIH, USA).

METHOD DETAILS

Yeast media

Cells were grown in YEP medium (1% yeast extract, 2% bactopeptone) supplemented with 2% glucose (YEPD), 2% raffinose (YEPR) or 2% raffinose and 3% galactose (YEPRG). Gene disruptions were generated by one-step PCR homology cassette amplification and standard yeast transformation method.

Search for rad50 mutants suppressing the sensitivity to CPT of sae2Δ cells

Genomic DNA from strains carrying the *KANMX* gene located 570 bp upstream of the *RAD50* ORF was used as template to amplify by low-fidelity PCR the *RAD50* coding region, respectively. Thirty independent PCR reaction mixtures were prepared, each containing 5U EuroTaq DNA polymerase (Euroclone), 10 ng genomic DNA, 500 ng each primer, 0.5 mM each dNTP (dATP, dTTP, dCTP), 0.1 mM dGTP, 0.5 mM MnCl2, 10 mM 2-mercaptoethanol, 10 mM Tris–HCl (pH9), 50 mM KCl and 1.5 mM MgCl2. The resulting PCR amplification products, containing the *RAD50* coding sequence and the *KANMX* resistance gene, respectively, were used to transform a *sae2Δ* strain. Three thousand transformants were selected and then assayed by drop tests for decreased sensitivity to high doses of CPT.

Spot assays

Cells grown overnight were diluted to 1×10^7 cells/ml. 10-fold serial dilutions were spotted on YEPD with or without indicated DNA damaging drugs. Plates were incubated for 3 days at 28°C.

Western blotting and immunoprecipitation

Protein extracts for western blot analysis were prepared by trichloroacetic acid (TCA) precipitation. Frozen cell pellets were resuspended in 200 μL 20% TCA. After the addition of acid-washed glass beads, the samples were vortexed for 10 min. The beads

were washed with 200 μL of 5% TCA twice, and the extract was collected in a new tube. The crude extract was precipitated by centrifugation at 3000 rpm for 10 min. TCA was discarded, and samples were resuspended in 70 μL 6X Laemmli buffer (60mM Tris, pH6.8, 2% SDS, 10% glycerol, 100mM DTT, 0.2% bromophenol blue) containing 0.9% 2-mercaptoethanol and 30 μL 1M Tris (pH8.0). Prior to loading, samples were boiled at 95°C and centrifuged at 3.000 rpm for 10 min. Supernatant containing the solubilized proteins were separated on 10% polyacrylamide gels. Rad53 was detected by using anti-Rad53 polyclonal antibodies (ab104232) from Abcam. Immunoprecipitations were performed as previously described (Cassani et al., 2018), with the following modifications: protein extracts were prepared in a lysis buffer containing 50 mM HEPES (pH 7.5), 140 mM NaCl, 1 mM EDTA (pH 7.5), 10% glycerol, 0.5% NP40, 1mM phenylmethylsulfonyl fluoride, 60 mM β-glycerophosphate, 1 mM sodium orthovanadate and a protease inhibitor cocktail (Roche Diagnostics).

Chromatin Immunoprecipitation and qPCR

ChIP analysis was performed as previously described (Cassani et al., 2016). Quantification of immunoprecipitated DNA was achieved by quantitative real-time PCR (qPCR) on a Bio-Rad CFX Connect Real-Time System apparatus. Triplicate samples in 20 μL reaction mixture containing 10 ng of template DNA, 300 nM for each primer, 2 × SsoFast EvaGreen® supermix (Bio-Rad #1725201) (2X reaction buffer with dNTPs, Sso7d-fusion polymerase, $MgCl_2$, EvaGreen dye, and stabilizers) were run in white 96-well PCR plates Multiplate (Bio-Rad #MLL9651). The qPCR program was as follows: step 1, 98°C for 2 min; step 2, 90°C for 5 s; step 3, 60°C for 15 s; step 4, return to step 2 and repeat 45 times. At the end of the cycling program, a melting program (from 65°C to 95°C with a 0.5°C increment every 5 s) was run to test the specificity of each qPCR. Data are expressed as fold enrichment at the HO-induced DSB over that at the non-cleaved *ARO1* locus, after normalization of each ChIP signals to the corresponding input for each time point. Fold enrichment was then normalized to the efficiency of DSB induction.

Preparation of recombinant proteins

The Mre11-Rad50 complex was expressed in *Spodoptera frugiperda 9* (*Sf9*) cells using constructs coding for His-tagged Mre11 and untagged Rad50, and purified by NiNTA (QIAGEN) affinity chromatography followed by ion-exchange chromatography with HiTrap SP HP (Cytiva) and HiTrap Q HP (Cytiva) columns (Oh et al., 2016). For the preparation of the Mre11-Rad50-N18S mutant complex, the construct for the expression of untagged Rad50 was mutagenized by site-specific mutagenesis with 5′-CCG CAG CTT CGA TAG CAG CGA TCG CGA GAC CAT CG-3′ and 5′-CGA TGG TCT CGC GAT CGC TGC TAT CGA AGC TGC GG-3′oligonucleotides. The mutant complex was purified using the same procedure as for the wild-type MR. Phosphorylated Sae2 was prepared in *Sf*9 cells as previously described (Cannavo et al., 2018). Recombinant Rif2 was prepared as a fusion with N-terminal MBP tag and a C-terminal 10xHis-tag in *Sf*9 cells. The pFB-MBP-Rif2-his construct was a kind gift from A. Bianchi (University of Sussex, UK). The cell extracts were prepared in 50 mM Tris-HCl pH 7.5, 1 mM dithiothreitol, 1 mM ethylenediaminetetraacetic acid, 1:400 Sigma protease inhibitory cocktail P8340, 1 mM phenylmethylsulfonyl fluoride, 30 μg/ml leupeptin, supplemented subsequently with 1/2 volume 50% glycerol and 6.5% volume 5 M NaCl. The soluble fraction was then incubated with amylose resin (New England Biolabs) and washed with MBP wash buffer I (50 mM Tris-HCl pH 7.5, 5 mM 2-mercaptoethanol, 1 M NaCl, 10% glycerol, 1 mM phenylmethylsulfonyl fluoride), followed by MBP wash buffer II (50 mM Tris-HCl pH 7.5, 5 mM 2-mercaptoethanol, 300 mM NaCl, 10% glycerol, 1 mM phenylmethylsulfonyl fluoride). The MBP fusion protein was eluted in MBP wash buffer II supplemented with 10 mM maltose, and the MBP tag was cleaved with PreScission protease (1:5, w/w, 2 h at 4°C). The sample was then supplemented with 10 mM imidazole and applied on NiNTA resin (QIAGEN). The resin was washed with NTA wash buffer A1 (50 mM Tris-HCl pH 7.5, 5 mM 2-mercaptoethanol, 1 M NaCl, 10% glycerol, 0.5 mM phenylmethylsulfonyl fluoride, 58 mM imidazole) and NTA wash buffer A2 (50 mM Tris-HCl pH 7.5, 5 mM 2-mercaptoethanol, 150 mM NaCl, 10% glycerol, 58 mM imidazole) and eluted with NTA buffer B (50 mM Tris-HCl pH 7.5, 5 mM 2-mercaptoethanol, 150 mM NaCl, 10% glycerol, 300 mM imidazole). Eluted Rif2-his protein was dialyzed into 50 mM Tris-HCl pH 7.5, 5 mM 2-mercaptoethanol, 150 mM NaCl, 10% glycerol, aliquoted, frozen in liquid nitrogen and stored at −80°C.

ATPase assays

The ATPase assays were carried out in 10 μL reactions in 25 mM Tris-acetate pH 7.5, 1 mM dithiothreitol, 5 mM magnesium acetate, 20 mM NaCl, 0.25 mg/ml bovine serum albumin (NEB), 150 μM unlabeled ATP, 4 nM γ-^{32}P-ATP (Perkin Elmer) and 200 ng of dsDNA. The reaction buffer was assembled on ice, the recombinant proteins were added, as indicated, and the reactions were incubated at 30°C for 4 h. The ATP hydrolysis was analyzed by thin layer chromatography using a standard procedure, the plates were exposed to storage phosphor screens and processed by a Typhoon Imager (GE Healthcare/Cytiva). The data were quantitated using ImageJ and plotted with Prism software.

Nuclease assays

Nuclease assays were carried out as previously described (Cannavo and Cejka, 2014; Cannavo et al., 2018) in 15 μL reactions in 25 mM Tris-acetate pH 7.5, 1 mM dithiothreitol, 5 mM magnesium acetate, 1 mM manganese acetate, 0.25 mg/ml bovine serum albumin (NEB), 1 mM phosphoenolpyruvate, 80 U/ml pyruvate kinase (Sigma), 1 mM ATP and 1 nM (in molecules) 3′-labeled dsDNA substrate (oligonucleotides PC1253C and PC1253B). Streptavidin (15 nM final, Sigma) was added to block one of the DNA ends, and the reactions were pre-incubated for 5 min at room temperature. Subsequently, where applicable, Rif2 was added and the reactions were incubated for 5 min at room temperature. The additional recombinant proteins (MR variants, phosphorylated Sae2, pSae2) were then added as

indicated, and the reactions were incubated for 30 min at 30°C. The reaction products were analyzed by denaturing electrophoresis, the gels were dried and exposed to storage phosphor screens and processed by a Typhoon Imager (GE Healthcare/Cytiva). The data were quantitated using ImageJ and plotted with Prism software.

Protein interaction assays

*Sf*9 cell lysate expressing MBP-Rif2-his was bound to amylose resin (NEB) and washed with wash buffer 1 M (50 mM Tris-HCl pH 7.5, 1 M NaCl, 0.2% [v/v] NP40, 2 mM EDTA, 1:1000 protease inhibitory cocktail (Sigma P8340) and then with wash buffer 0.1 M (same as wash buffer 1 M, but only 0.1 M NaCl). The recombinant MR variants (1 µg) were then added, incubated for 1 h at 4°C and the resin was then washed with wash buffer 0.1 M. As a negative control, the MR proteins were added to the amylose resin without MBP-Rif2-his to test for non-specific binding. The proteins were eluted with 0.1 M wash buffer supplemented with 20 mM maltose, and cleaved with PreScission protease. The eluates were analyzed by Ponceau staining or by western blotting with anti-His (MBL, D291-3, 1:5,000) or anti-Rad50 antibodies (Thermo Scientific, PA5-32176, 1:1,000).

QUANTIFICATION AND STATISTICAL ANALYSIS

Data are expressed as mean values ± SD or SEM. Quantification and statistical analysis were done using PRISM (GraphPad). *p* values for the ChIP-qPCR and recombination experiments were calculated by two-tailed Student's t test. No statistical methods or criteria were used to estimate sample size or to include or exclude samples.

Cell Reports, Volume *34*

Supplemental information

Sae2 and Rif2 regulate MRX endonuclease activity at DNA double-strand breaks in opposite manners

Antonio Marsella, Elisa Gobbini, Corinne Cassani, Renata Tisi, Elda Cannavo, Giordano Reginato, Petr Cejka, and Maria Pia Longhese

SUPPLEMENTAL INFORMATION

Table S1, related to STAR methods. *Saccharomyces cerevisiae* strains used in this study.

Strain	Relevant genotype	Source
W303	*MAT*a/α *ade2-1 can1-100 his3-11,15 leu2-3,112 trp1-1 ura3-1 rad5-535*	
YLL 1134.2	W303 *rif2*Δ*::KANMX*	Cassani et al., 2016
DMP 6166/2D	W303 *MAT*α *rif2*Δ*::HIS3 sae2*Δ*::KANMX*	This study
YLL 1069.3	W303 *sae2*Δ*::KANMX*	Cassani et al., 2018
HS21	*MATα ade5-1 his7-2 ura3Δ trp1-289 leu2-3,112::p305L3 LEU2 lys2::AluIR*	Lobachev et al., 2002
YLL 4197.1	HS21 *rif2*Δ*::TRP1*	This study
YLL 3773.3	HS21 *sae2*Δ*::KANMX*	This study
YLL 4348.1	HS21 *mre11-H15N::LEU2*	This study
DMP 7294/6A	HS21 *rif2*Δ*::TRP1 sae2*Δ*::KANMX*	This study
YLL 4349.2	HS21 *mre11-H15N::LEU2 rif2*Δ*::TRP1*	This study
JKM139	*MAT*a *hml*Δ*::ADE1, hmr*Δ*::ADE1*, *ade1-100, lys5, leu2-3,112, trp1::hisG ura3-52, ho, ade3::GAL-HO site*	Lee et al., 1998
YLL 1712.6	JKM139 *rif2*Δ*::HPHMX*	This study
DMP 5113/5A	JKM139 *KANMX::rad50-N18S*	This study
YLL 3627.3	JKM139 *sae2*Δ*::HPHMX*	This study
DMP 7144/8C	JKM139 *rif2*Δ*::KANMX sae2*Δ*::HPHMX*	This study
YLL 3757.1	JKM139 *KANMX::rad50-N18S sae2*Δ*::HPHMX*	This study
YLL 1854.2	JKM139 *MRE11-18MYC::TRP1*	Cassani et al., 2018
DMP 6149/2D	JKM139 *sae2*Δ*::KANMX MRE11-18MYC::TRP1*	This study
DMP 7248/3A	JKM139 *rif2*Δ*::KANMX sae2*Δ*::HPHMX MRE11-18MYC::TRP1*	This study
DMP 6211/5B	JKM139 *rif2*Δ*::HPHMX MRE11-18MYC::TRP1*	This study
YLL 3222.6	JKM139 *TEL1-3HA::NATMX*	Cassani et al., 2018
DMP 6435/1A	JKM139 *sae2*Δ*::KANMX TEL1-3HA::NATMX*	This study
DMP 7250/8D	JKM139 *sae2*Δ*::HPHMX rif2*Δ*::KANMX TEL1-3HA::NATMX*	This study
DMP 7250/8A	JKM139 *rif2*Δ*::KANMX TEL1-3HA::NATMX*	This study
YLL 3421.2	JKM139 *RAD9-3HA::TRP1*	This study
DMP 7192/6B	JKM139 *sae2*Δ*::HPHMX RAD9-3HA::TRP1*	This study
DMP 7192/8C	JKM139 *sae2*Δ*::HPHMX rif2*Δ*::KANMX RAD9-3HA::TRP1*	This study
DMP 7192/3A	JKM139 *rif2*Δ*::KANMX RAD9-3HA::TRP1*	This study
YLL 4104.2	JKM139 *MRE11-H125N-18MYC::TRP1*	This study

DMP 7283/3D	JKM139 *rif2Δ::KANMX MRE11-H125N-18MYC::TRP1*	This study
YLL 3754.1	W303 *KANMX::rad50-N18S sae2Δ::HIS3*	This study
DMP 7007/8B	W303 *KANMX::rad50-N18S sae2Δ::HIS3 rif2Δ::HIS3*	This study
DMP 6449/1C	W303 *KANMX::rad50-N18S*	This study
YLL 941.1	W303 *yku70Δ::HIS3*	This study
DMP 4374/6B	W303 *sae2Δ::KANMX yku70Δ::HIS3*	This study
DMP 6903/18D	W303 *KANMX::rad50-N18S ykuΔ::HIS3*	This study
DMP 6903/7D	W303 *KANMX::rad50-N18S sae2Δ::KANMX yku70Δ::HIS3*	This study
DMP 6903/9A	W303 *KANMX::rad50-N18S sae2Δ::KANMX yku70Δ::HIS3*	This study
DMP 7238/2A	JKM139 *KANMX::rad50-N18S MRE11-18MYC::TRP1*	This study
DMP 7238/7A	JKM139 *KANMX::rad50-N18S sae2Δ::HPHMX MRE11-18MYC::TRP1*	This study
DMP 7238/7A	JKM139 *KANMX::rad50-N18S sae2Δ::HPHMX rif2Δ::KANMX MRE11-18MYC::TRP1*	This study
YLL 3612.2	JKM139 *RIF2-18MYC::URA3*	This study
DMP 7059/2A	JKM139 *RIF2-18MYC::URA3 KANMX::rad50-N18S*	This study

Figure S1

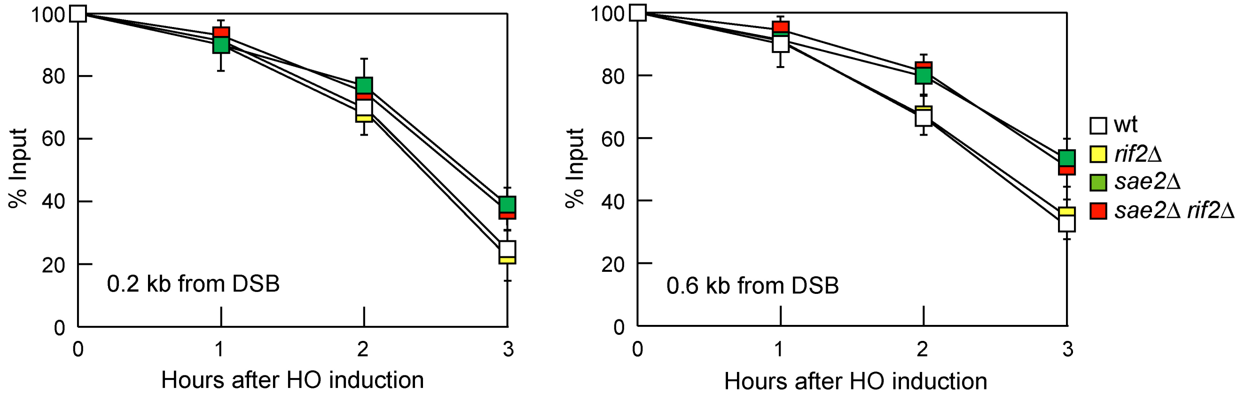

Figure S1, related to Figure 3A. Kinetics of input degradation at the HO-induced DSB. qPCR on input DNA at the indicated time points after HO induction (time zero). qPCR values at the HO-induced DSB were normalized to the corresponding values at the non-cleaved *ARO1* locus for each time points. Data are expressed relative to time zero that was set up at 100%. The mean values of three independent experiments are represented with error bars denoting s.d.
